# Global distribution of a chlorophyll *f* cyanobacterial marker

**DOI:** 10.1038/s41396-020-0670-y

**Published:** 2020-05-26

**Authors:** Laura A. Antonaru, Tanai Cardona, Anthony W. D. Larkum, Dennis J. Nürnberg

**Affiliations:** 1 Department of Life Sciences, Imperial College, London, UK; 2 Global Climate Cluster, University of Technology Sydney, Sydney, NSW Australia; 3 Institute for Experimental Physics, Freie Universität Berlin, Berlin, Germany

**Keywords:** Environmental microbiology, Microbial ecology

## Abstract

Some cyanobacteria use light outside the visible spectrum for oxygenic photosynthesis. The far-red light (FRL) region is made accessible through a complex acclimation process that involves the formation of new phycobilisomes and photosystems containing chlorophyll *f*. Diverse cyanobacteria ranging from unicellular to branched-filamentous forms show this response. These organisms have been isolated from shaded environments such as microbial mats, soil, rock, and stromatolites. However, the full spread of chlorophyll *f*-containing species in nature is still unknown. Currently, discovering new chlorophyll *f* cyanobacteria involves lengthy incubation times under selective far-red light. We have used a marker gene to detect chlorophyll *f* organisms in environmental samples and metagenomic data. This marker, *apcE2*, encodes a phycobilisome linker associated with FRL-photosynthesis. By focusing on a far-red motif within the sequence, degenerate PCR and BLAST searches can effectively discriminate against the normal chlorophyll *a*-associated *apcE*. Even short recovered sequences carry enough information for phylogenetic placement. Markers of chlorophyll *f* photosynthesis were found in metagenomic datasets from diverse environments around the globe, including cyanobacterial symbionts, hypersaline lakes, corals, and the Arctic/Antarctic regions. This additional information enabled higher phylogenetic resolution supporting the hypothesis that vertical descent, as opposed to horizontal gene transfer, is largely responsible for this phenotype’s distribution.

## Introduction

Oxygenic photosynthesis powers the vast majority of life on Earth. In this process, chlorophyll pigments capture solar energy and store it into chemical bonds. The main pigment is chlorophyll *a*. Substituted variants like chlorophyll *b* and divinyl chlorophylls have evolved to expand the absorption within the visible spectrum [1]. For a long time, it was thought that the visible spectrum is the absolute limit for oxygenic photosynthesis, with longer-wavelength photons being too low in energy for water splitting.

Discovered in 2010 from stromatolites in Shark Bay (Australia), chlorophyll *f* is a form of chlorophyll that enables a subset of cyanobacteria to photosynthesize in far-red light (FRL) [2]. In a complex acclimation process called FaRLiP (Far-Red Light Photoacclimation), chlorophyll *f* replaces ~10% of a cell’s chlorophyll *a* molecules [3], these include the primary electron donors in both photosystems and a small number of longer-wavelength antenna [4]. Plants and other oxygenic photosynthetic organisms generally absorb visible light, with little to no absorption beyond ~700 nm, thereby creating FRL-rich environments in their shade, in which these cyanobacteria can live [5].

The FRL (>700 nm) reaching the Earth surface is abundant, containing approximately a fifth of the photons of PAR (400–700 nm, photosynthetically active radiation) [6]. However, the contribution of FRL to the biosphere, in terms of energy input, is unknown. This could be significant in environments where energy sources and light are limited such as hot spring mats, and desert rocks and soil. One way to understand energy input is to look at distribution. Understanding the natural conditions in which these cyanobacteria thrive could help highlight their capabilities and limits, but only a few strains have been discovered until now.

Chlorophyll *f*-producing organisms have been isolated from various shaded environments such as microbial mats, soil, and stromatolites [2, 7–9]. However, relatively little is known of their full spread in nature. Although some of the first chlorophyll *f*-containing cyanobacteria were identified biochemically [10, 11], the majority of strains has been recovered through bioinformatics, by identifying a gene cluster associated with far-red photoacclimation in sequenced genomes [3]. A subset of them was later tested for experimental proof of chlorophyll *f* formation [7]. In addition, a limited number of far-red cyanobacteria were recovered by growing samples from FRL-rich areas, such as caves, lakes, and soil under selective FRL, including the first known chlorophyll *f* species, *Halomicronema hongdechloris* [2, 11–14]. There have even been reports of chlorophyll *f* being directly detected in cyanobacterial mats [15], beach rock [16], and other intertidal rocks [17]. A recent study focusing on subtropical forests [9] has indicated that the far-red niche might be more common and diverse than previously assumed.

Testing samples under FRL is the main limiting step when trying to identify new chlorophyll *f*-producing cyanobacteria. It is a time-consuming, and in the case of environmental samples, an especially tedious process. Contamination with other organisms is a problem. Culture-based tests also necessarily ignore the microbial dark matter, the vast majority of Earth’s bacteria that cannot be cultured [18].

In comparison, much more is known about the environmental spread of *Acaryochloris marina*, the only other cyanobacterial species known to photosynthesize in FRL. It does so by using a majority of chlorophyll *d* (>90%) [19]. Chlorophyll *d* is the only other known red-shifted chlorophyll. Methods based on detecting chlorophyll *d* directly (HPLC) [20, 21] or phylogenetic proxies involving a specific 16S rRNA marker [22] have revealed a global spread of this cyanobacterium with its habitat ranging from polar to tropical regions. The use of the 16S rRNA gene as a marker has been especially favorable. Not only has it enabled the accurate identification of *A. marina* through PCR from smaller samples than pigment-based methods and using less specialized equipment, but also it opens up the possibility of finding *A. marina* in metagenomic sequencing surveys.

In far-red-light acclimated cells, chlorophyll *f* represents ~10% of the total chlorophyll in the cell, as opposed to more than 90% chlorophyll *d* in *A. marina* [6, 7, 10]. This makes pigment-based identification less sensitive. Moreover, while photosynthesis using a chlorophyll *d* majority seems to be restricted to *A. marina* [22], chlorophyll *f*-producing cyanobacteria are widely distributed across most major clades [3, 5, 7, 8, 15]. They are spread among the five traditional phylogenetic/morphological Sections within the Cyanobacteria phylum, and closely related species often differ in their ability to photosynthesize in FRL. This wide distribution means that a simple phylogenetic marker such as the 16S rRNA cannot be used.

Here we developed a method to identify chlorophyll *f*-containing cyanobacteria using a marker gene. BLAST searches in genomic and metagenomic data highlight this marker in a variety of environments across the world, from the Atacama Desert to the Antarctic, while primers targeting the marker have helped us to recover far-red strains from environmental samples. The new sequences provide a better understanding of the evolution of this unusual adaptation. Phylogenetic trees indicate that far-red photosynthesis is an ancient adaptation with vertical descent and loss playing a dominant role in its current distribution.

## Materials and methods

### Cyanobacterial strains and environmental samples

Cyanobacterial strains were obtained from the NIES (Japan), PCC (France), and SAG (Germany) culture collections and are listed in Table S1. Strains not previously known for far-red acclimation were identified through bioinformatics (see “Primer design”).

Additional strains were recovered from environmental samples. Beach rock samples were collected in July 2016 from Heron Island (Australia) from the black beach rock area as previously described [16]. Small pieces of thrombolites were sampled from Lake Clifton (Australia) in June 2016 (for further details on the sampling site see [23, 24]).

### Culture conditions

All strains were grown in BG11 media [25] at room temperature except for *Calothrix parasitica* NIES-267(f/2 marine medium) [26], *Hydrococcus rivularis* NIES-593 (CB medium) [27], and *Acaryochloris marina* (K + ESM) [28].

Environmental samples (beach rock and thrombolites) were enriched in chlorophyll *f*-containing cyanobacteria by growing them under 750 nm light (Epitex, L750-01AU) at room temperature in liquid or on solid media (1.5% (w/v) agar). The Clifton thrombolite isolate of *Halomicronema* was isolated by repeated reinoculation in BG11 media containing 33 and 66 g/l NaCl. Beach rock isolates were grown in BG11 containing 0 (“beach rock 4”), 16.5 (“beach rock 2”), or 33 g/l NaCl (“beach rock 5”). To reduce fungal contamination 50 µg/ml cycloheximide was added.

### DNA extraction

Genomic DNA from cyanobacterial cultures and environmental samples was extracted using Quick-DNA Fungal/Bacterial Miniprep Kit (Zymo Research). Beach rock and thrombolite material was homogenized with a mortar and pestle prior to extraction.

### Primer design

The FaRLiP cluster consists of ~20 genes in different orientations and arrangements [3, 7], and therefore an effective PCR marker for chlorophyll *f* photosynthesis would not span more than one gene. The region amplified should ideally carry enough information for phylogenetic assignment, making large genes the likeliest candidates. The genes *rfpA* (red-shifted phytochrome, ~2650 bp), *apcE2* (phycobilisome linker protein, ~2270 bp), and *psaA2* (photosystem I subunit, ~2355 bp) were therefore considered, alongside the smaller chlorophyll *f* synthase gene *psbA4* (~1130 bp). Far-red-associated homologues were recovered through BLAST searches and assembled into multiple alignments (protein and DNA) which were then visually inspected.

Multiple alignments (Clustal Omega, ten HMM iterations) [29] were performed on these genes using Seaview software [30]. Consequent phylogenies showed that the putative far-red sequences form monophyletic groups. The alignments, both protein and DNA, were then inspected in order to find stretches of ~20 bp that were conserved among the “far-red” sequences, but distinct from the “standard” ApcE/PsaA/RfpA-like paralogs. The more conserved ApcE set was considered more suitable for this task. Alignments were visualized with Seaview [30] and Jalview [31].

The ApcE2 sequence motif VIPEDV was identified as unique marker for selection (see Results). This motif corresponds to amino acid numbers 204 to 209 (in *Chroococcidiopsis thermalis*). It represents the forward primer target.

20 bp tags were added at the 5′ end of the primers to improve product recovery [32]. The initial tags are predicted to have low homology to cyanobacterial DNA as evaluated by BLAST searches. The alternative forward tag is loosely based on the upstream sequence of the forward primer’s target [33]. Annealing temperatures were matched with Multiple Primer Analyzer (Thermo Fisher Scientific).

### PCR

PCR was performed using Q5 HF polymerase (NEB) and 25 µg genomic DNA in 25 µl reactions. Following initial denaturation (98 °C, 30 s), there were 30 cycles of: denaturation (98 °C, 10 s), annealing (58 °C, 20 s), and expansion (72 °C, 45 s). Final extension step: 72 °C, 2 min. PCR products were separated by gel electrophoresis, extracted with a QIAquick Gel Extraction Kit (Qiagen), and sequenced (Eurofins). Chromatograms were viewed with DNA Baser (Heracle BioSoft) and UGENE [34].

For beach rock samples 4 and 5, there was an additional cloning step to recover multiple *apcE2* (or 16S rRNA) variants, as their original sequencing chromatograms showed overlapping bases, hinting at multiple strains. The blunt-ended PCR products were subjected to poly-A tailing and ligated into a pGEM-T vector (Promega). Plasmids were extracted with a Monarch Plasmid Miniprep Kit (NEB) and 5–15 colonies/gene/sample were sequenced.

### Absorbance spectra

UV–Vis spectra from cultures grown in far-red and white light were taken with a UV-1601 spectrophotometer (Shimadzu).

### Confocal microscopy

Fluorescence images and spectral scans of cyanobacterial samples were acquired with a Leica SP5 inverted laser scanning confocal microscope using a ×63 objective. Samples were immobilized by spotting cells from liquid media onto growth media-agar (1.5 %) pads. Samples were excited at 488 nm and fluorescence emission was recorded from 650–680 nm for phycobilisomes and chlorophyll *a* and 720–750 nm for chlorophyll *d*/*f*. In addition, spectral emission scans were performed from 620–800 nm using the Leica lambda scan application wizard. Images were analyzed with Leica LAS AF Lite and FIJI [35] and spectra were plotted with OriginPro (OriginLab) software.

### Pigment analysis

Pigments were extracted and analyzed by HPLC as previously described [4].

### Metagenomic data analysis

To retrieve additional FaRLiP strains from (meta)genomic data several approaches were used. Firstly, the *C. thermalis* ApcE2 sequence was used as a query in BLAST searches. Databases searched: Genbank Whole Genome Shotgun (WGS) [36], JGI/MER metagenomics database [37]. WGS settings: tblastn; target group: cyanobacteria, accessed 04/2018. JGI/MER settings: assembled metagenomes, removing those including the terms “human”/“gut”/“intestin”, accessed 07/2018.

Alignments of BLAST hits were checked for the FaRLiP-related VIPEDV-like motif. The matching sequences were recovered in both DNA and protein format. Identical sequences were removed.

Secondly, a 46 amino acid fragment of the *C. thermalis* ApcE2 containing the VIPEDV motif was used to search for gene fragments. A short fragment could match short (100 bp) unassembled reads. In JGI/MER, both assembled and unassembled metagenomes were searched.

To search a subset of the NCBI SRA, the same fragment was used as a query on the SearchSRA website [38] (www.searchsra.org). Top hits were checked with ExPASy Translate [39] and confirmed with BLAST.

### Phylogeny

Phylogenies were built with PhyML in Seaview [30, 40] and with RaxML on the CIPRES webserver [41, 42]. PhyML settings: model GTR, aLRT (SH-like), empirical equilibrium frequencies, invariable sites optimized, best of NNI and SPR, five random starts. RaxML settings: RAxML-HPC2 on XSEDE, model GTR GAMMA, bootstrap iterations 1000. Trees were edited with iTOL [43] and Inkscape 0.92. The root point for *apcE2* trees was selected to match previous ApcE phylogenies [7, 44].

## Results

### Identification of a marker for chlorophyll *f* photosynthesis: apcE2

Seven new strains of cyanobacteria with putative far-red-associated genes were recovered by BLAST searches of far-red genes *apcE2* and *rfpA*, in addition to 11 known or suspected far-red strains with sequenced genomes. They include three *Fischerella* strains (NIES-4106, NIES-592, and NIES-3754), two *Calothrix* strains (NIES-267 and NIES-3974), cyanobacterium TDX16, and *Hydrococcus rivularis* NIES-593. As with all known far-red strains, their FaRLiP cluster was located on the genome, except for *Fischerella sp*. NIES-4106, where it was found on a megaplasmid (~312 kb).

These additional sequences helped with building primers. For a gene fragment to be used as a PCR marker, it would have to be conserved among far-red strains while being distinct from white-light sequences. One such conserved “far-red island” was found in the large gene *apcE2*. Potential PCR primer targets in *rfpA, psaA2*, and *psbA4* were shorter and showed poor sequence conservation. Therefore, a set of primers was developed for *apcE2*, a gene which encodes the peptide connecting the phycobilisome antenna to the thylakoid membrane [45]. It should be noted that in a genomic context, *apcE2* has always and exclusively been found in far-red (FaRLiP) clusters, which always contain *psbA4*, the chlorophyll *f* synthase gene [3, 7, 46]. The use of *apcE2* as a marker for far-red cyanobacteria has been suggested before based on *apcE2* sequences forming a distinct clade from *apcE1*, as well as on the lack of a relevant cysteine [5, 7]. Furthermore, it was recently reported that *apcE2* knock-out mutants failed to grow under FRL despite the active synthesis of chlorophyll *f* [47].

**Fig. 1:**
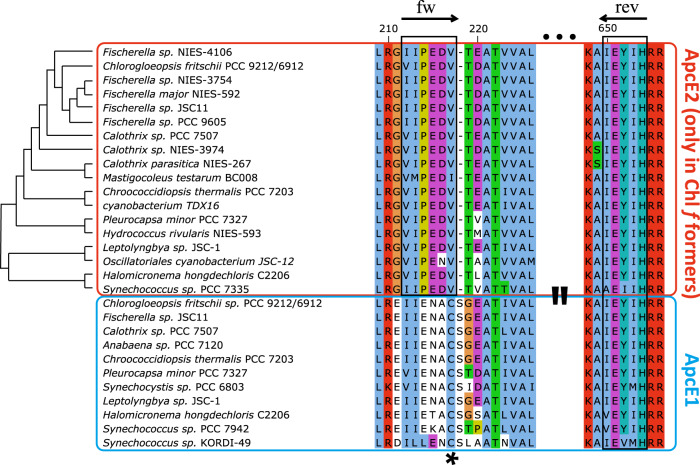
Conserved far-red specific motifs are present in ApcE2 fragments. Outlined in red are ApcE2 sequences associated with the far-red cluster, including close homologues found in this study. A phylogeny of the full-length DNA sequences is shown on the left-hand side and in Fig. 5 in more detail. The blue box encompasses conventional (white-light) ApcE sequences. Conventional ApcE is found in chlorophyll *f*-containing organisms, along far-red ApcE2. The phytochrome-binding cysteine (residue 217, asterisk) is missing in far-red sequences, and instead they show a conserved VIPEDV-like motif (residues 204–209 in *C. thermalis*). Black boxes and arrows highlight areas covered by the primers designed in this study, both in a far-red-specific area (fw) and a conserved ApcE-specific area (rev).

Our reverse primer targets an area universal to all *apcE* genes (Fig. 1). The forward primer targets an area (~210 bp from the start of the gene) associated with chromophore binding that shows conservation at both protein and DNA level within *apcE2* (far-red associated) sequences but is distinct from white-light *apcE1* versions. This is the case even when compared to *apcE1* sequences from early-branching strains such as the marine *Synechococcus* (e.g., strain KORDI-49) (Figs. 1, S1). At the protein level, far-red sequences (known or suspected) contain a highly conserved VIPEDV-like motif, while white-light ApcE1 proteins contain an ENACS-like motif (Fig. S2).

The missing cysteine in ApcE2 (residue 217, Fig. 1) relates to an important shift in function. The non-covalent binding of phycocyanobilin in the far-red ApcE2 leads to a red-shift in the absorbance (700 nm in the far-red *Synechococcus sp*. PCC 7335, as opposed to 660 nm for covalent binding) [7, 48, 49]. It seems likely that such a shift to long wavelengths would only be beneficial to far-red organisms. As such, this primer set tests the presence of a gene fragment directly connected to a far-red physiological function.

Three sets of primers of varying degeneracy values were designed (Table S2). They included a variable 3′ section as well as a non-variable 5′ tag. The primer pair that gave the highest level of amplification across various species was used in the following experiments (Table 1).

**Table 1:** Main sets of primers used in this study.

^a^*f* forward, *r* reverse. *M* refers to medium degeneracy in comparison with other primers tested in this study (Table S2). *t* represents tags. Asterisks mark versions of the same primer with an alternate tag.

^b^All degenerate primers had 5′ tags. Red boxes mark any base (*N*); blue boxes mark bases with a degeneracy of 3 (H = not G; D = not C; V = not T); green boxes mark bases with a low degeneracy (Y = C/T; R = A/G).

**Fig. 2:**
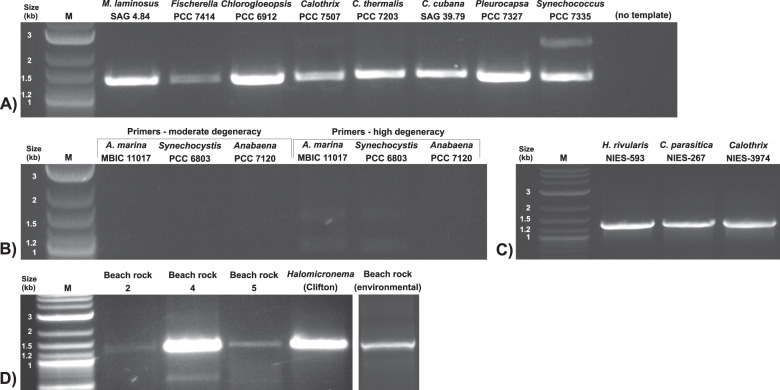
a*pcE2* is a marker of chlorophyll *f*. *apcE2* primers are specific for chlorophyll *f-*forming cyanobacteria, and the amplicons they form can be seen as ~1.2 kb bands on agarose gels. They can be used to efficiently distinguish between (**a**) strains that have the far-red acclimation cluster and (**b**) strains that lack it. Primers of moderate degeneracy were used unless otherwise mentioned. Even at higher degeneracy, no false positives appeared in the negative controls. **c** Newly-sequenced strains from the Japanese NIES culture collection tested positive for *apcE2*. The gene was originally found via BLAST searches. **d** Particularly significant, the method is useful for discovering new strains. The *apcE2* gene is present in enriched samples from Heron Island beach rock (“beach rock 2,4,5”) and thrombolites from Lake Clifton (*Halomicronema*, Clifton). It was also recovered directly from a beach rock environmental sample (rightmost, separate PCR). The environmental sample band is the result of three consecutive PCR runs; however, a faint band was visible after the first run. This strongly suggests that chlorophyll *f* cyanobacteria are present in these environments. Molecular weight marker: 2-Log Ladder.

The primers were tested on a set of 14 species (Table S2, Fig. 2). These include cyanobacteria previously proven to produce chlorophyll *f* (*Chroococcidiopsis thermalis* PCC 7203, *Chlorogloeopsis fritschii* PCC 6912, *Calothrix sp*. PCC 7507, *Synechococcus sp*. PCC 7335) [7, 50], as well as strains predicted to be capable of far-red-light acclimation (*Fischerella muscicola* PCC 7414, *Pleurocapsa sp*. PCC 7327) due to the presence of the cluster [3]. Two additional strains were predicted as likely to have the cluster based on phylogeny. *Mastigocladus laminosus* SAG 4.84 is phylogenetically similar to *F. muscicola* PCC 7414 [51] and *Chroococcidiopsis cubana* SAG 39.79 is related to *C. thermalis* PCC 7203 [52]. All predicted strains were grown under FRL and produced far-red chlorophylls as indicated by the shift in absorption (Fig. S3).

PCR products appeared as bands at ~1.2 kb for samples known or expected to contain the *apcE2* gene (Table S1, Fig. 2a). Their identity was confirmed through sequencing. No amplification was seen in negative controls. These included commonly-used model strains such as *Anabaena sp*. PCC 7120 and *Synechocystis sp*. PCC 6803 and the chlorophyll *d*-containing cyanobacterium *A. marina* MBIC-11017 (Fig. 2b). The primer set with the highest degeneracy (Table S2) showed faint nonspecific amplification in both negative and positive samples, but only the positive samples showed a clear 1.2 kb band.

Three additional strains from the NIES culture collection, highlighted in this study as potential chlorophyll *f*-formers, were also investigated (Fig. 2c). Overall, the strains predicted to give a positive result are a diverse assortment covering four out of the five cyanobacterial Sections, from the unicellular *Synechococcus sp*. PCC 7335 to complex cyanobacteria such as the branched, heterocyst-forming *M. laminosus*.

**Fig. 3:**
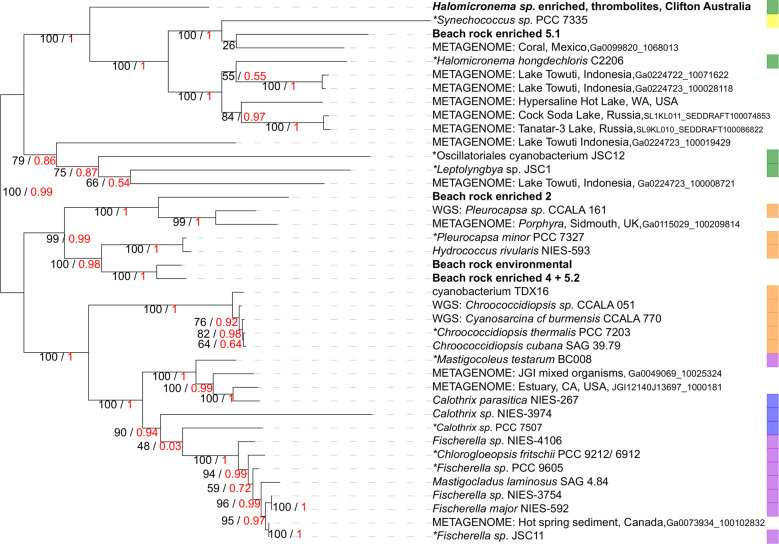
Phylogenetic tree reconstruction of *apcE2* variants illustrates previously unknown diversity. The tree was built with PhyML (red, aLRT values) and RaxML (black, 1000 bootstrap). With the given root point, this gene tree resembles a cyanobacterial species tree. The analysis includes sequences recovered from metagenomics databases (labeled as “metagenome”) or the whole-genome-sequencing NCBI database (WGS). This gene is present in a diverse array of far-red photosynthesizing cyanobacteria, including unicellular (Section I, labeled yellow), aggregates (Section II, orange), filamentous (Section III, green), heterocyst-forming (Section IV, blue) as well as branched and heterocyst-producing forms (Section V, purple). Sequences from the beach rock strains (2, 4, 5.1, and 5.2) and the *Halomicronema* Clifton isolate were recovered experimentally. Asterisks mark strains associated with FR acclimation before this study.

Interestingly, the phylogeny of *apcE2* sequences (Fig. 3) has a highly similar pattern to that inferred from large 16S rRNA phylogenies [53] (also see Fig. S4), multigene phylogenies [54, 55], and *apcE1* phylogenies (Fig. S5). The amplified fragments alone appear to carry sufficient information for accurate phylogenetic placement. *Synechococcus sp*. PCC 7335, *Halomicronema*, and the Oscillatoriales cluster together, with their ancestral sequence branching out early from the rest. Heterocyst-forming cyanobacteria cluster together in both *apcE2* and species trees, with *Chroococcidiopsis* as a sister clade. Not only are these large, Section-level distinctions clear in the *apcE2* tree, but the finer details of the phylogenies are also remarkably similar (such as within the *Fischerella* genus) (Figs. 3, S4).

### New chlorophyll *f* cyanobacteria from far-red light rich environments

The beach rock environment has been previously shown to contain chlorophyll *f-*producing cyanobacteria [16]. It represents an aggregation of sand and silt, often biological in nature, which hosts a rich endolithic photosynthetic community [56, 57]. As such, beach rock samples from Heron Island (Australia) were collected to test the *apcE2* primers, in addition to thrombolites from Lake Clifton (Australia). Thrombolites are also structures formed by cyanobacteria, similar in shape and morphogenesis to stromatolites, from which the chlorophyll *f* cyanobacterium *Halomicronema hongdechloris* has been isolated [2, 24, 58].

The primers were tested on genomic DNA recovered from crushed beach rock containing endolithic cyanobacteria. The amplified sequence matched a *Pleurocapsa*-like *apcE2* in BLAST searches (76% identity; labeled as “Beach rock, environmental” in Fig. 2d). The primers were also tested on enriched samples, where beach rock or thrombolite fragments had been grown under FRL (Fig. 2d). These cultures had been inoculated in media with different salt concentrations (0–66 g/l) to recover multiple strains.

A *Halomicronema*-like fragment of *apcE2* was recovered from the Clifton thrombolite enrichment. This is supported by the 16S rRNA sequence similarity and the observed morphology. Formation of chlorophyll *f* results in a shift in fluorescence [3, 4]. In combination with microscopy, this can be a powerful tool to distinguish far-red-photosynthesizing cyanobacteria. Fluorescence micrographs show the production of red-shifted pigments with a maximal fluorescence emission at around 740–760 nm when grown under FRL (Fig. S6A). Pigment extraction followed by HPLC analysis confirmed the presence of chlorophyll *f* (Fig. S6B).

**Fig. 4:**
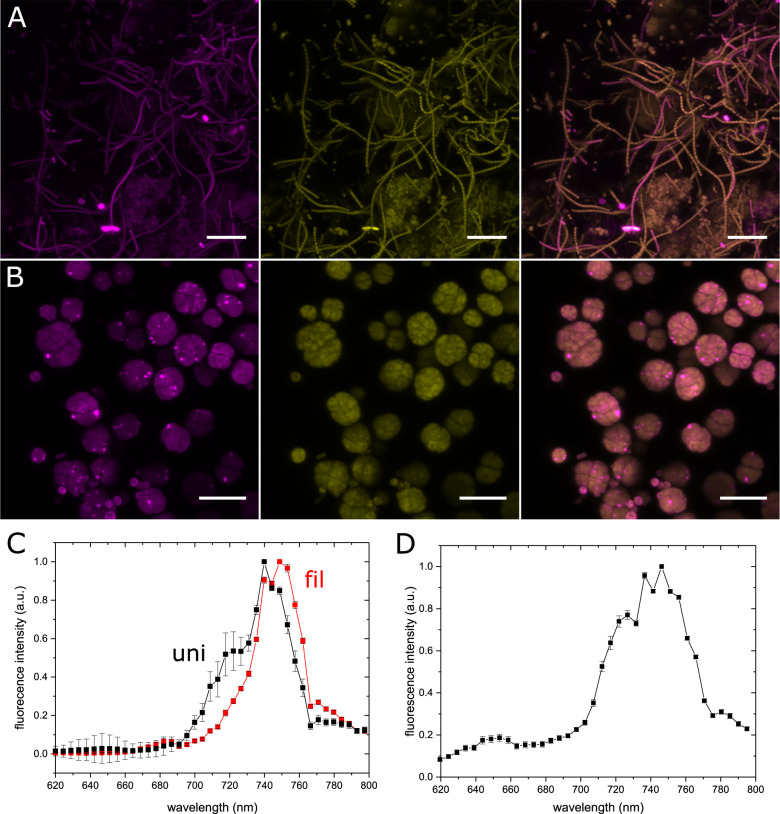
Confocal imaging of cyanobacteria from beach rock enrichment samples. Sample “beach rock 5” (**a**) contains unicellular and filamentous strains. Genetic data suggests the presence of a filamentous *Halomicronema*, aggregate-forming *Pleurocapsa* and unicellular *Acaryochloris*. Sample “beach rock 2” (**b**) represents a relatively pure culture of aggregate-forming cyanobacteria similar to *Pleurocapsa*. The strains in the image contain chlorophyll *a* and phycobilisomes (magenta, fluorescence emission range 650–680 nm), but also chlorophyll *d/f* (yellow, 720–750 nm). See right-hand column for overlay. Scale bar 15 μm (**b**) and 25 µm (**a**). Spectral scans of individual cells shown in (**a**) are displayed in (**c**). Cells were divided in filamentous (fil, black) and unicellular forms (uni, red). The spectral scans of cells shown in (**b**) are given in (**d**). The error bars indicate the difference in fluorescence between individual cells relative to the maximum intensity.

Out of three beach rock enrichment samples, all showed the shift in fluorescence to longer wavelengths indicative for the presence of far-red pigments (Fig. 4). Also, all showed the 1.2 kb band when tested with the primers (Fig. 2d, Table S2). However, only one of the bands gave a high-quality sequencing result (“beach rock 2”). For samples “beach rock 4” and “beach rock 5”, the chromatograms showed overlapping bases, suggesting a mixture of sequences. To recover individual *apcE2* fragments, the PCR products were cloned into plasmids that were individually sequenced. “Beach rock 4” produced multiple sequences closely related to *Pleurocapsa minor* PCC 7327, out of which the most common was used for phylogenetic analysis. “Beach rock 5” showed a *Pleurocapsa*-like sequence (“beach rock 5.2”) and a *Halomicronema*-like sequence (“beach rock 5.1”). Representative sequences are shown in the *apcE2* phylogeny in Fig. 3.

Cloning was also used to recover 16S rRNA fragments from the diverse community thriving in the enriched media (Fig. S4, RaxML 1000 bootstrap phylogeny). For beach rock samples 2 and 4, the 16S rRNA sequences clustered with *Pleurocapsa* and with the closely-related, non-FaRLiP cyanobacterium *Cyanothece sp*. PCC 8801. This largely matches the results from the *apcE2* phylogeny. For sample “beach rock 5”, one 16S rRNA fragment recovered was similar to the *Pleurocapsa* sequence, fitting similar *apcE2* results. However, the 16S rRNA for the filamentous, far-red strain in this sample proved more elusive. No *Halomicronema*-like sequence was recovered. Instead, we observed multiple sequences more (98%) or less (95%) related to *Acaryochloris marina*. Pigment extraction and HPLC studies on this sample revealed a high (>10%) percentage of chlorophyll *d*, likely confirming the presence of *Acaryochloris* in the sample. It is nevertheless possible that one of the sequences (e.g., 95% identity to *Acaryochloris*, 94% identity to filamentous *Leptolyngbya*) could belong to a filamentous chlorophyll *f* producer.

There was only one 16S rRNA variant recovered from the Clifton sample. It had 98% identity to *H. hongdechloris* 16S rRNA and the two sequences clustered together in the 16S rRNA tree (Fig. S4), confirming the identity of the isolate as a close relative.

### Metagenomic data analysis indicate a wide distribution of chlorophyll *f*-forming cyanobacteria

ApcE2 (far-red) and ApcE1 (white-light) sequences fall into distinct clades [7] (Fig. S1). However, there was no clear difference in sequence similarity values between ApcE2 and ApcE1 in BLAST searches. Occasionally, very divergent sequences (e.g., *Synechococcus sp*. PCC 7335) were labeled as less similar to certain ApcE2 versions than some ApcE1 proteins. Therefore, using short FRL-specific motifs helps with exploring sequence databases. This is especially important when the genomic context might be fragmented, contaminated, or incomplete (e.g., metagenomics).

Sixteen new sequences were found by blasting the GenBank WGS database [36]. Thirteen of them are *Fischerella* sequences (25 isolates). They are highly similar to known sequences like *Fischerella* JSC-11 and as such not further discussed (Table S4). The three other sequences correspond to the groups of *Pleurocapsa* and *Chroococcidiopsis*.

**Fig. 5:**
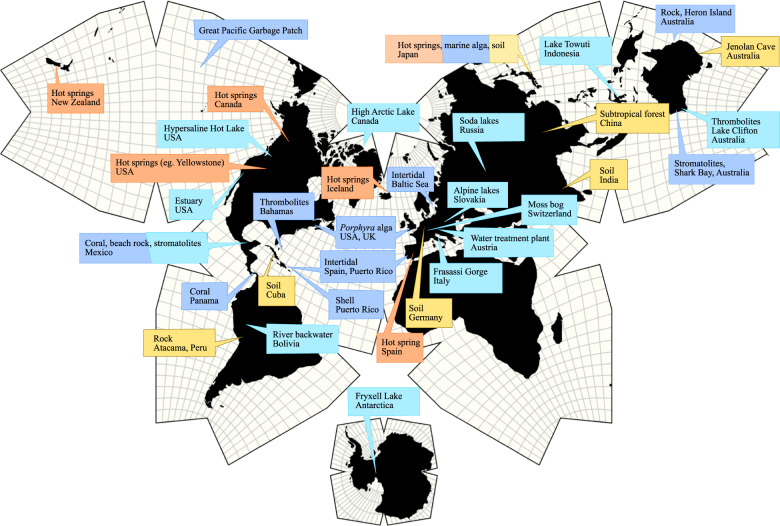
Chlorophyll *f*-containing cyanobacteria are distibuted in a variety of environments across the globe. The illustration is based on previous pigment and genomic data as well as recent metagenomic and environmental data. Locations are color-coded based on environment. Dark blue: marine. Red: hot springs. Yellow: terrestrial. Light blue: terrestrial aquatic (including a wide range from oligotrophic to hypersaline to nutrient-rich).

In addition, blasting 14,716 assembled metagenomes in the JGI/MER database [37] uncovered ten new full-length ApcE2-like sequences. Short fragments were not initially considered. In *apcE2* phylogenies, they represent a diverse array covering four of the five cyanobacterial Sections (assuming the strains they were isolated from are similar in morphology to their sister clades). The locations from which the samples were collected range from hypersaline, high-pH lakes, to oligotrophic freshwater lakes, to associations with other organisms (Fig. 5, Table S3).

The number of environments in which chlorophyll *f* plays a role increases even further if gene fragments are considered. The search query in this case was a 46 amino acid sequence centered on the VIPEDV motif. The databases searched included the JGI database, as well as the NCBI Sequence Read Archive. Fragments of far-red *apcE2* were found in extreme environments such as the Antarctic, the Arctic, and the Atacama Desert, as well as in anthropogenic environments such as a wastewater treatment plant and the Pacific Garbage Patch (Figs. 5, S7, Table S4). There were no far-red sequences recovered from the TARA Oceans project data [59].

## Discussion

The developed PCR-based method with degenerate primers is an efficient technique for uncovering new chlorophyll *f*-producing cyanobacteria. The primers efficiently distinguish against the conventional *apcE1* gene by targeting a FR-specific motif. Nevertheless, it is plausible that there might be cases where *apcE2* is not associated with chlorophyll *f*. For example, as pseudogenes or in cyanobacteria using other, as-of-yet unknown red-shifted chlorophylls or adaptations. However, our method represents a fast, relatively cheap assay that works with environmental samples as well as cultured strains. Furthermore, the sequences recovered from amplicons are sufficient for accurate phylogenetic placement. Best results were obtained by partially constraining degeneracy (“primers of moderate degeneracy”), but in theory, more degenerate primers might be able to amplify unusually divergent sequences that this set could miss. Although false positive (amplification without chlorophyll *f*) and negatives (chlorophyll *f* present, but no amplification) could exist, none were observed during the course of our experiments. Therefore, we suggest that this method could greatly speed up the pace of discovery in the field. Further research could expand knowledge on the spread of chlorophyll *f* photosynthesis in nature, both at macro-(geographical) and microscales (e.g., within a single cyanobacterial mat).

One such environment is represented by beach rock. Beach rock is a complex cyanobacterial-rich environment [56]. Considerable alterations in salinity, light intensity, and water availability occur with the changing of tides [56, 57]. In our beach rock samples, we have found a variety of chlorophyll *f*-containing cyanobacteria. These include baeocystous (aggregate-forming) strains related to *Pleurocapsa*, and a filamentous *Halomicronema*-like strain. *Acaryochloris*, the unicellular chlorophyll *d* producer, was also present. A previous paper identified in beach rock an additional far-red strain morphologically similar to *Chroococcidiopsis* [16]. This highlights that the far-red niche within beach rock is not a “far-red desert” but a rich, diverse environment. It is interesting to ask whether certain strains have a preference for specific microenvironments within beach rock, and whether other environments have a comparable FR species richness.

Another environment tested was thrombolites. Thrombolites are microbialites, structures formed by microbes through the cementation of sand and minerals [24]. They are similar to stromatolites, from which the first chlorophyll *f* organism (*H. hongdechloris*) was isolated. Fragments of far-red *apcE2* with a VIPEDV motif were also found in shotgun metagenomic data from previous thrombolite and stromatolite studies [23, 60] (Table S6).

Our bioinformatic analysis has greatly expanded the range of known chlorophyll *f* organisms (see Table S7 for GenBank accessions). Gene trees built with RaxML were largely congruent between *apcE2* and 16S rRNA. This hints at chlorophyll *f* being an ancient adaptation.

As sequencing becomes cheaper, genomic and metagenomic data has increased exponentially [61, 62]. Mining metagenomic data can lead to insights, not only about the overall abundance of a trait in nature, but also about the environments in which it may be found, community composition, and the evolution of chlorophyll *f* photosynthesis. For example, our data suggests that a *Halomicronema*-like species might be especially abundant in microbial mats in the hypersaline Hot Lake, Washington, USA. Out of 12 sequenced biosamples reported on the JGI GOLD website, 11 contained a FR-specific, *Halomicronema*-like *apcE2* (the remaining one contained a fragment). This is consistent with a previous study on Hot Lake biofilms reporting cyanobacteria living below a depth of 1.5 mm, where <1% of photons at 440 and 665 nm are able to reach [63]. In this shaded environment, usage of near-infrared light by phototrophs was noted. We suggest that chlorophyll *f* photosynthesis might partially explain this data, along with the presence of bacteriochlorophyll-containing species.

Shading in the visible spectrum might also occur in the specific case of symbiosis and close associations. *Calothrix parasitica* NIES-267 was isolated from within a red alga. Closely related to it in the phylogeny is the taxon labeled “JGI mixed organisms”, which contains sequence data from host-associated organisms recalcitrant to axenic growth. An *apcE2* gene with homology to *Pleurocapsa* was found in the metagenome of the red alga *Porphyra*, collected from the intertidal area in Sidmouth (UK). This fits with previous observations of cyanobacteria from the order Pleurocapsales forming biofilms on the holdfast (attachment point) of this alga [64].

Previously, *Acaryochloris marina*, a far-red cyanobacterium producing chlorophyll *d*, was also found as an epiphyte on red algae [21, 65], and it is likely that a similar epiphyte is responsible for the original discovery of chlorophyll *d* in red algal extracts [66]. Red algae contain pigments, such as phycoerythrin and phycocyanin, that absorb mostly in the blue-to-yellow area of the spectrum, leaving FRL to pass through [67]. This could provide a resource for far-red cyanobacteria, but only close to the surface, as FRL is efficiently absorbed by the water column itself [68]. The lack of *apcE2* sequences in the Tara Ocean data [59] further supports this. As more strains become available, it would be interesting to study whether symbiosis is a common evolutionary choice for FR cyanobacteria.

The ability to search short (~100 bp), unassembled reads for markers of chlorophyll *f* photosynthesis is important, because databases like the NCBI SRA contain a huge wealth of sequence data (ca. 10k terabases at the time of writing). This can further expand the limits of what was known of far-red acclimation and identify new niches. For example, this study is the first to highlight a potential chlorophyll *f* marker in the Arctic and Antarctic. There are multiple far-red sequences from samples in the Pacific Garbage Patch, unusual in the sense that chlorophyll *f* photosynthesis has never been reported in the ocean mass. However, the Pacific Garbage Patch is an anthropogenic gathering of microplastics that has been described as a “plastic soup”. The authors of the respective metagenomic study have mentioned the presence of more biofilm-forming cyanobacteria, as opposed to planktonic [69], supporting the idea that the plastic serves as “rafts” for the formation of distinct plastisphere communities [70].

Fragmented sequences can also help highlight the richness of far-red habitats. For example, ten of the environments discovered to contain far-red *apcE2* fragments, contained sequences that could be attributed to more than one cyanobacterial group. This includes algae sampled across the Atlantic Ocean (showing homology to *Pleurocapsa* and the obligate marine *Synechococcus sp*. PCC 7335), bacterial mats from Chocolate Pots, Yellowstone (showing homology to filamentous non-heterocystous Oscillatoriales as well as heterocyst-forming *Fischerella*), and varied microbialite samples from across the world. The distribution is consistent with recent results showing a high diversity of far-red cyanobacteria in a subtropical forest [9].

The evolution of the far-red acclimation is still poorly understood. It has been suggested to be an ancient innovation [15], but the evidence for it is scant. It appears likely that different genes entered the far-red cluster at different points in evolutionary time. The gene duplication leading to *psbA4*, the chlorophyll *f* synthase [71], for instance, is likely to predate the most recent common ancestor of known cyanobacteria [72]. For this reason, it has been suggested that chlorophyll *f* may even predate oxygenic photosynthesis [71]. However, this is contradicted by research which argues that water photooxidation preceded not only the split between *psbA4* (“super-rogue”) and other *psbA* (D1) genes, but also the split between photosynthetic components D1 (*psbA)* and D2 *(psbD)* [73–75].

Horizontal gene transfer was suggested as the most likely mechanism to explain the spread of chlorophyll *f* photosynthesis across distant genera, with the exception of heterocyst-forming cyanobacteria [7]. Phylogenetic trees from multiple far-red genes were similar, suggesting limited horizontal gene transfer at single-gene level [7]. However, the additional sequence data in our study suggest that vertical descent and loss could play a more important role than previously considered, e. g., in groups such as the Pleurocapsales and *Halomicronema*. Given the current data, all present-day clusters could be explained without cluster-level horizontal transfer events.

We could not find unambiguous evidence of horizontal gene transfer of an entire FaRLiP gene cluster in our data. The sequence for *Synechococcus sp*. PCC 7335 seems to branch out from within the *Halomicronema* clade, which appears inconsistent with the species tree. However, this could be an artefact caused by an apparent faster rate of evolution of the *Synechococcus* sp. PCC 7335 sequence leading to long-branch attraction. Alternatively, the strain (identified morphologically) might need to be reclassified using molecular sequence data [76].

The similar topologies between the 16S/23S rRNA and the ApcE2 trees are most evident in Section IV and V (heterocystous and branched-heterocystous) cyanobacteria as previously mentioned [7]. However, it also extends to other groups, including *Chroococcidiopsis*, the sister group of heterocyst-forming cyanobacteria [77]. This is strongly consistent with vertical descent and indicates that repeated losses of FaRLiP gene clusters has certainly occurred within at least these groups (see Fig. S1). Given that the split between the heterocystous cyanobacteria and their closest non-heterocystous relatives is dated to ~1.6 billion years ago [78] based on molecular clocks, though younger dates have been suggested [55], the far-red cluster might be older than this. Therefore, FaRLiP may have originated during the late Paleoproterozoic/early Mesoproterozoic, when stromatolites reached their greatest diversity and distribution, followed by widespread losses coincidental with their decline in the Neoproterozoic/Phanerozoic [79]. Nevertheless, the rise of eukaryotes and the emergence of modern-day terrestrial environments opened new niches where this adaptation has endured.

More far-red sequences exist in metagenomics databases, for instance in the unassembled metagenomes not considered in this study. Doubtlessly more still will be discovered as more sequences are deposited online. It is plausible that FRL contributes more to primary production in tidal and terrestrial regions than previously assumed, both in moderate and extreme environments.

## Supplementary information

Supplemental material
