# Correction to: Global distribution of a chlorophyll *f* cyanobacterial marker

**DOI:** 10.1038/s41396-022-01205-y

**Published:** 2022-02-15

**Authors:** Laura A. Antonaru, Tanai Cardona, Anthony W. D. Larkum, Dennis J. Nürnberg

**Affiliations:** 1 Department of Life Sciences, Imperial College, London, UK; 2 Global Climate Cluster, University of Technology Sydney, Sydney, NSW Australia; 3 Institute for Experimental Physics, Freie Universität Berlin, Berlin, Germany

**Keywords:** Environmental microbiology, Microbial ecology

Erratum to: ISME J. 2020:14:2275–87 10.1038/s41396-020-0670-y, published online 26 May 2020

Following the publication of this article, the authors noted errors regarding the sequences and lengths of the primers f_apcE2t* and f_apcE2M* listed in Table 1 and Table S2. In addition, the colors of the highlighted bases were missing in Table 1.

**Table 1:**
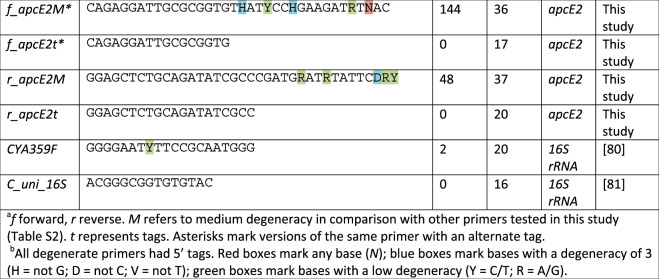
Main sets of primers used in this study.

The original article and the Supplementary Information have been corrected.

## Supplementary information

Supplemental material

