# Supplementary material for: Global distribution of a chlorophyll f cyanobacterial marker
Source: ISME J. 2020 May 26;14(9):2275–87. doi: 10.1038/s41396-020-0670-y (PMC7608106; doi:10.1038/s41396-020-0670-y)
Supplement: Supplementary file 1 — Supplemental material [file 41396_2020_670_MOESM1_ESM.docx]

**Supplementary data**

|  | | High degeneracy primers | Medium degeneracy primers | Medium degeneracy primers + alternate tag | Low degeneracy primers | |
| --- | --- | --- | --- | --- | --- | --- |
| *Mastigocladus laminosus* SAG 4.84 | | + | ++ | ++ | +++ | |
| *Fischerella sp.* PCC 7414 | | (+) | (+) | + | ++ | |
| *Chlorogloeopsis sp.* PCC 6912 | | ++ | +++ | +++ | ++ | |
| *Calothrix sp.* 7507 | | ++ | + | ++ | + | |
| *Chroococcidiopsis thermalis* PCC 7203 | | (+) | + | ++ | (+) | |
| *Chroococcidiopsis cubana* SAG 39.79 | | (+) | + | ++ | (+) | |
| *Pleurocapsa minor* PCC 7327 | | ++ | ++ | ++ | +++ | |
| *Synechococcus sp.* PCC 7335 | | +++ | + | + | + | |
| *Calothrix sp.* NIES-3974 | |  |  | * |  | |
| *Calothrix parasitica* NIES-267 | |  |  | * |  | |
| *Hydrococcus rivularis* NIES-593 | |  |  | * |  | |
| Beach rock 2 | |  |  | * |  | |
| Beach rock 4 | |  |  | * |  | |
| Beach rock 5 | |  |  | * |  | |
| Beach rock, environmental | |  |  | * |  | |
| *Halomicronema*, Clifton | |  |  | * |  | |
| *Synechocystis sp.* PCC 6803 | | 0 |  | 0 |  | |
| *Anabaena sp.* PCC 7120 | | 0 |  | 0 |  | |
| *Acaryochloris marina* MBIC-11017 | | 0 |  | 0 |  | |
| **Table S1.** Efficiency of product formation with *apcE2*-specific primers. The amount of product was estimated from band intensity on 0.8% agarose gels, in FIJI software, using the 3 kb band of the 2-Log Ladder for calibration. 0: no product; *: product present, amount unspecified; (+): less than 100 ng product; +: 100 to 200 ng; ++: 200 to 400 ng; +++: above 400 ng product. 25 ng gDNA per 25 µl of reaction. High degeneracy primers often showed non-specific amplification along the main bands. The primers with moderate degeneracy and an alternate forward tag are the most consistent in getting high yields across multiple FR species. | | | | |  |  |

| **Primer^a^** | **Sequence (5’ to 3’)^b^** | **Degeneracy** | **Length (nt)** | **Gene** | **Ref.** |
| --- | --- | --- | --- | --- | --- |
| *f_apcE2H* | GCCGGAGCTCTGCAGATATCTNATNCCNGAARATRTNAC | 1024 | 39 | *apcE2* | This study |
| *f_apcE2M* | GCCGGAGCTCTGCAGATATCTHATYCCHGAAGATRTNAC | 144 | 39 | *apcE2* | This study |
| *f_apcE2M** | CAGAGGATTGCGCGGTGTHATYCCHGAAGATRTNAC | 144 | 36 | *apcE2* | This study |
| *f_apcE2L* | GCCGGAGCTCTGCAGATATCTHATYCCHGAAGATGTVAC | 54 | 39 | *apcE2* | This study |
| *f_apcE2t* | GCCGGAGCTCTGCAGATATC | 0 | 20 | *apcE2* | (1) |
| *f_apcE2t** | CAGAGGATTGCGCGGTG | 0 | 17 | *apcE2* | This study |
| *r_apcE2H* | GGAGCTCTGCAGATATCGCCCGATGDATRWWYTCDRY | 576 | 37 | *apcE2* | This study |
| *r_apcE2M* | GGAGCTCTGCAGATATCGCCCGATGRATRTATTCDRY | 48 | 37 | *apcE2* | This study |
| *r_apcE2L* | GGAGCTCTGCAGATATCGCCCGATGRATRTATTCRAT | 8 | 37 | *apcE2* | This study |
| *r_apcE2t* | GGAGCTCTGCAGATATCGCC | 0 | 20 | *apcE2* | This study |
| *CYA359F* | GGGGAATYTTCCGCAATGGG | 2 | 20 | *16S rRNA* | (2) |
| *C_uni_16S* | ACGGGCGGTGTGTAC | 0 | 16 | *16S rRNA* | (3) |

**Table S2.** Full set of primers tested in this study. f – forward; r – reverse. H, M or L refer to ‘high’, ‘medium’ and ‘low’ in terms of degeneracy. t represents tags. Asterisks mark versions of the same primer with an alternate tag. ^b^All degenerate primers had 5’ tags. Red boxes mark any base (N); blue boxes mark bases with a degeneracy of 3 (H=not G; D=not C; V=not T); green boxes mark bases with a low degeneracy (Y=C/T; R=A/G; W=A/T).

All primers had 5’ tags (1). These non-degenerate sequences improved primer-template binding (and therefore product recovery), and were used for sequencing. Tag-less primers produced no visible bands on agarose gels. Sequencing results were low-quality when using the forward tag; therefore, an alternate forward tag was developed. It was adapted from the consensus sequence upstream of the primer target sequence, similar to the CODEHOP method (4). This improved the quality of the sequencing results.

The ‘high degeneracy primers’ contained all the possible combinations of nucleotides found in the multiple alignment at specific positions (degeneracy 1024 (forward); 576 (reverse)). In contrast, primers of low degeneracy (54 (forward); 8 (reverse)) ignored nucleotides that appeared only once at certain positions. Between them, primers of moderate degeneracy (144 (forward); 48 (reverse)) ignored one-off bases, provided they did not occur in the 5 bp closest to the 3’ end. Previous research has shown that mismatches in the 5 bp closest to the 3’ end greatly diminish the chances of successful PCR amplification (5).

**
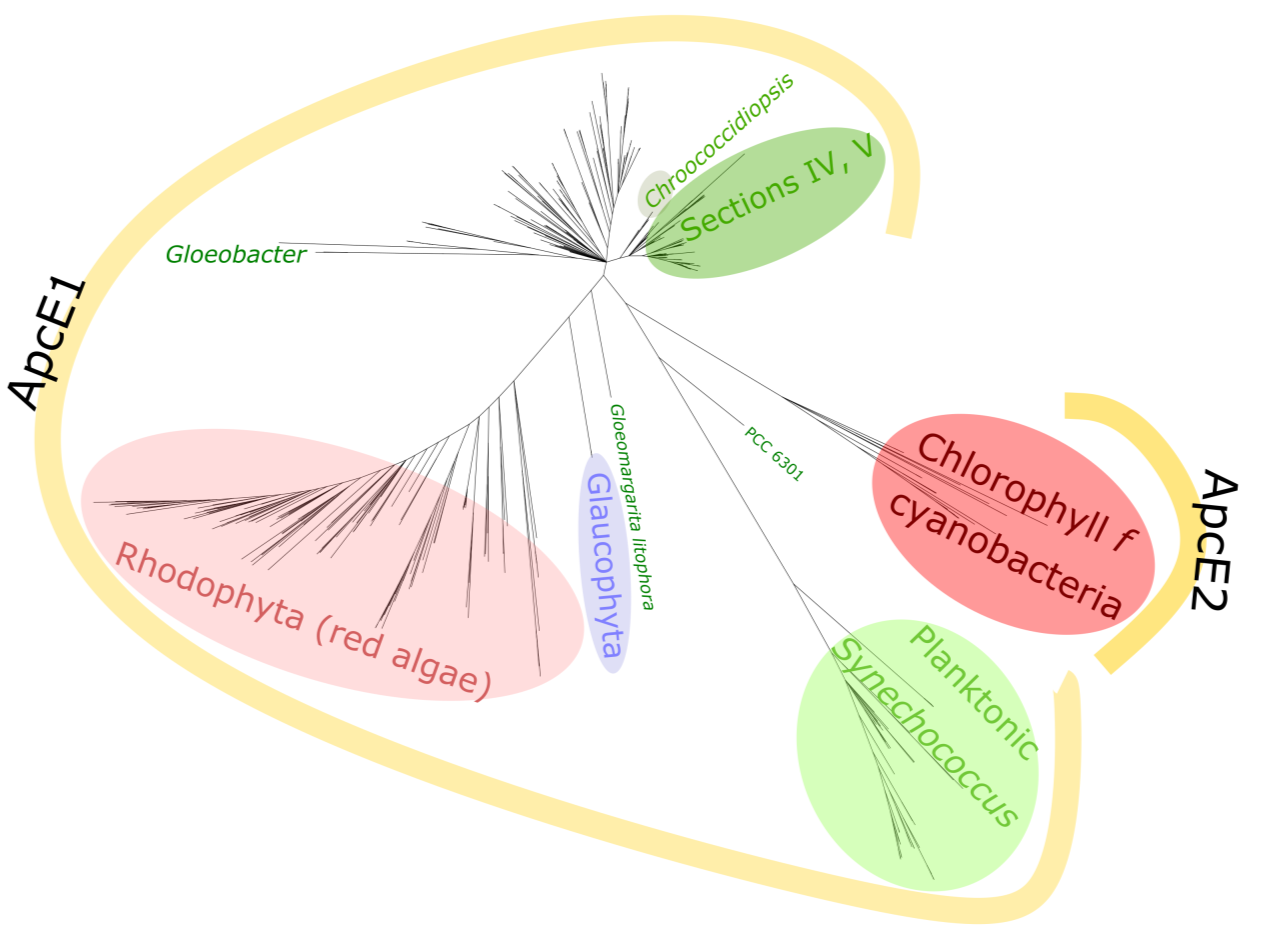
**

**Figure S1.** Protein tree of 489 ApcE sequences. ApcE2 sequences cluster together and are characteristic only of chlorophyll *f*-containing cyanobacteria, which also contain conventional ApcE1 copies. Far-red (ApcE2) sequences form a monophyletic clade and appear to represent a sister clade to ApcE in the early-branching planktonic *Synechococcus* group (including PCC 6301). Note the similarities with the *Synechecoccus sp*. KORDI-49 sequence in Figure 1. The emergence of this (largely marine) group is suggested to have occurred after the Great Oxygenation Event, with estimations ranging from 2.35 to 1.25 billion years ago (6-8). This would tentatively place chlorophyll *f-*based photosynthesis before this time point.

Phycobilisome component ApcE (also known as L_CM_, core-membrane linker) is present in cyanobacteria (bright red for ApcE2, green for ApcE1) as well as algae (purple for glaucophytes, light red for rhodophytes)(9). *Gloeomargarita litophora* is hypothesized to be the sister-clade to the chloroplast lineage (10). *Gloeobacter* was chosen as a root point. Sequences recovered with BLAST. MUSCLE alignment. Phylogeny executed with RaxML-HPC2 on XSEDE, GTRCAT (due to the large number of sequences), 1000 bootstrap, and illustrated with iTOL and Inkscape. Branches with <50 bootstrap support are collapsed. Similar results were obtained by removing highly divergent columns from the alignment (GBLOCKS).

**
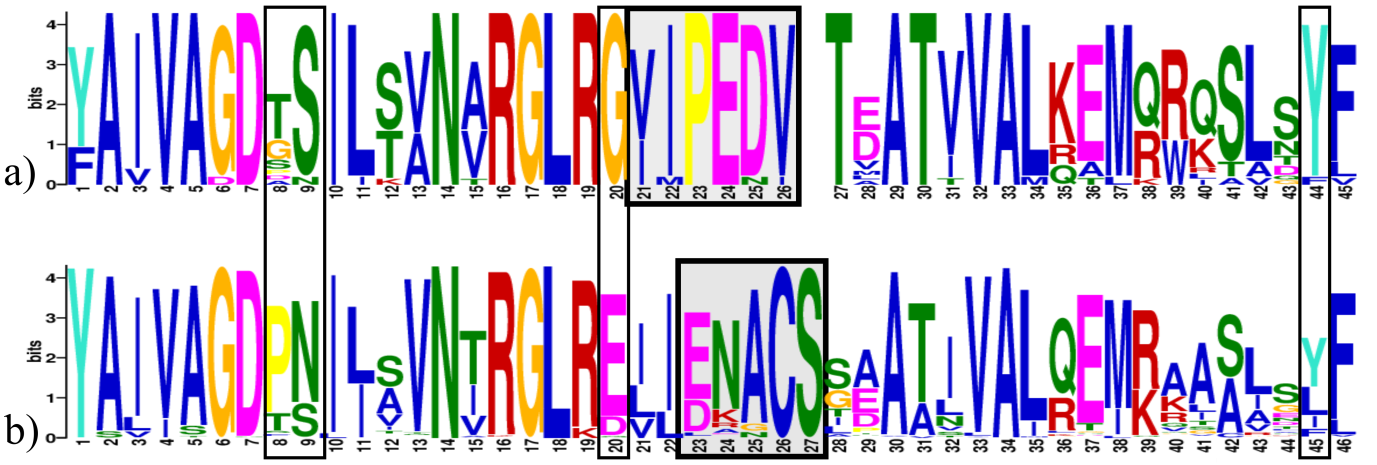
**

**Figure S2.** Extended motifs surrounding the chromophore-binding area in the chlorophyll *f*-associated marker ApcE2 (a), compared to motifs in white-light ApcE1 (b). The most distinguishable difference is the VIPEDV motif in far-red ApcE2, as opposed to ENACS in ApcE1 (grey boxes). This relates to non-covalent versus covalent chromophore binding.

However, within +/-20 amino acids from these motifs, there also exist subtler distinctions between ApcE1 and ApcE2. This involves certain amino acids being more commonly encountered in one paralogue than in the other. For example, proline is common at position 8 in the white-light sequences, but nearly never occurs in far-red ones. Tyrosine is almost always present at position 44 in far-red sequences, but it is less conserved among the white-light ones. This expanded motif made searching unassembled metagenomics databases possible.

18 ApcE2 sequences were used for the motif in a). 470 ApcE1 sequences were used for b). Figure created using the MEME webserver (11).


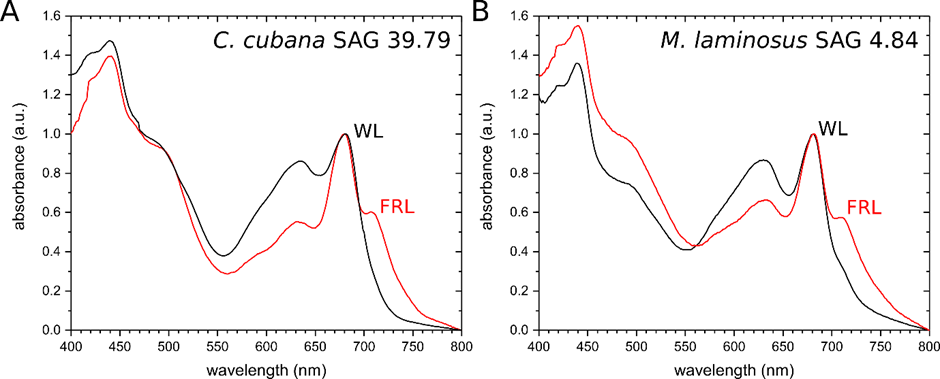


**Figure S3.** Absorption spectra of cells of *Chroococcidiopsis cubana* SAG 39.79 (A) and *Mastigocladus laminosus* SAG 4.84 (B) grown under white light (WL, black) and far-red light (FRL, red). The additional peak at 707 nm has been attributed to the formation of chlorophyll *d* and *f* (e.g. Nürnberg et al., 2018).

**
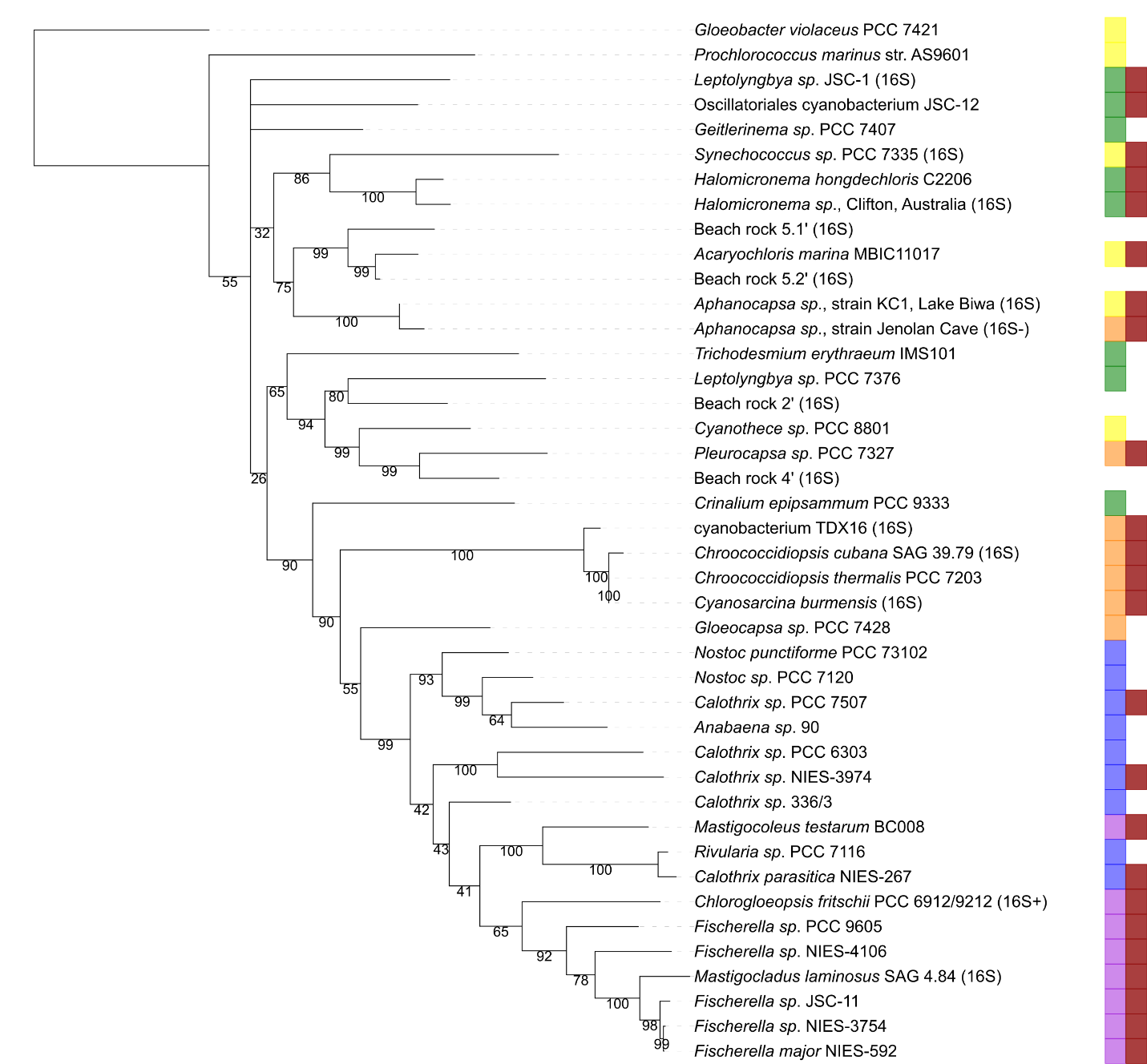
**

**Figure S4.** A 16S+23S rRNA phylogeny of a cyanobacterial subset approximating the species tree. This includes strains known or suspected to photosynthesize in far-red light (marked in dark red) which have very diverse morphologies. Yellow: unicellular; orange: baeocystous; green: filamentous; blue: filamentous heterocyst-forming; purple: branched-filamentous, heterocyst-forming. MUSCLE alignment, GBLOCKS. RaxML, 1000 bootstrap, GTRGAMMA. *Gloeobacter* as rootpoint. For some strains, the 23S sequence was not available and they were labelled as such (e.g. ‘16S+’ for sequences including more than the 16S rRNA, but not the 23S; ‘16S’ for sequences of the length amplified by commonly used primers).


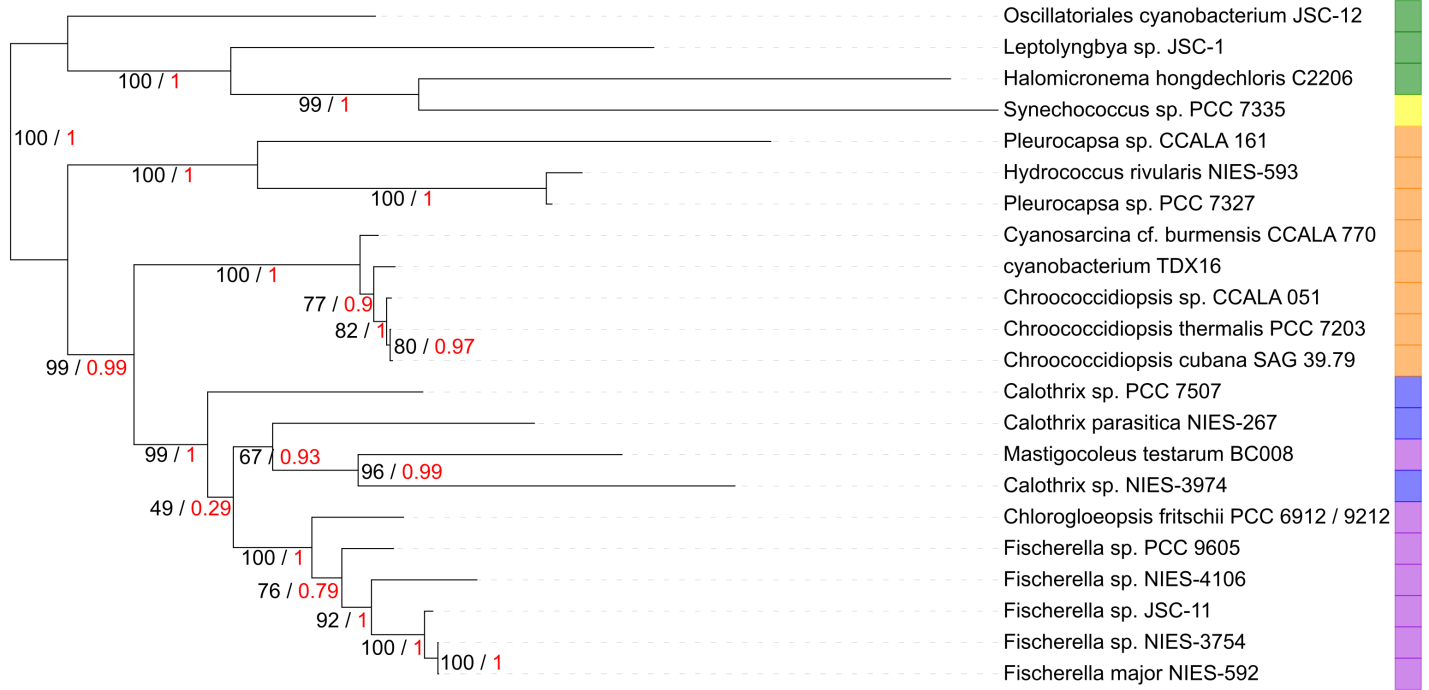


**Figure S5.** Phylogenetic tree reconstruction of *apcE1* variants executed with PhyML (red, aLRT values) and RaxML (black, 1000 bootstrap). This gene is present in all cyanobacteria, including unicellular (Section I, labelled yellow), aggregates (Section II, orange), filamentous (Section III, green), heterocyst-forming (Section IV, blue) as well as branched and heterocyst-producing forms (Section V, purple). Only chlorophyll-*f*-containing strains are shown here for comparison purposes. The tree is very similar to both the *apcE2* gene tree (Figure 3) and the 16S+23S tree above (Figure S4), suggesting shared evolutionary paths.


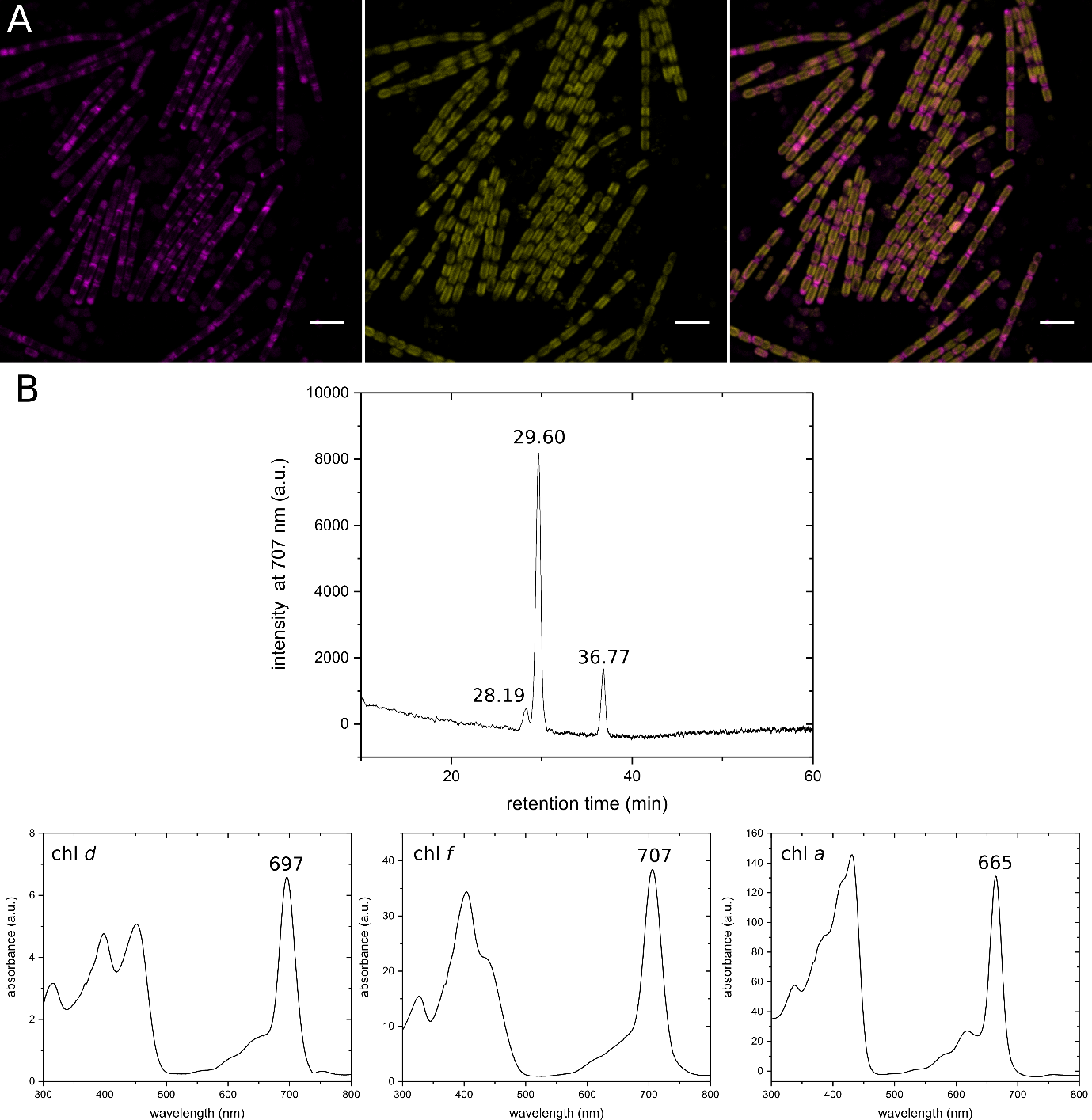


**Figure S6.** *Halomicronema-*enriched sample from Lake Clifton. (A) Confocal micrographs showing (from left to right) chlorophyll *a* and phycobilisome fluorescence (magenta; emission range: 670-700 nm), chlorophyll *f* fluorescence (yellow; emission range: 720-750 nm) and an overlay image. Scale bars, 5 µm. (B) HPLC analysis of isolated pigments indicates the presence of chlorophyll *a* (at 36.77 min), *d* (at 28.19 min) and *f* (at 29.60 min) as indicated by their absorption spectra.


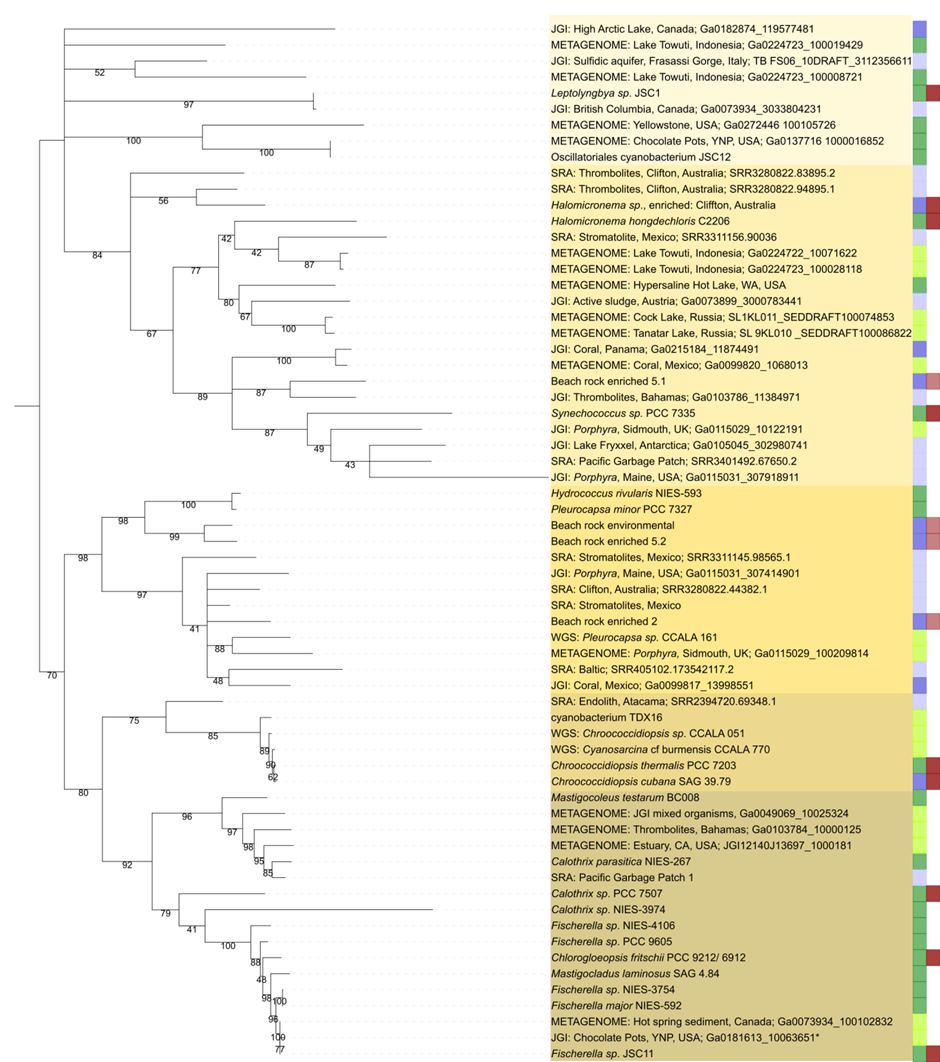


**Figure S7.** Phylogeny of *apcE2* sequences and fragments from genomic and metagenomic databases. This significantly increases the number of environments where chlorophyll *f* is present. The dataset includes sequences that are associated with a full far-red cluster (dark green), sequences associated with a partial cluster (lime green), fragments >300bp (dark blue) and short, <300bp fragments (light blue). It is worth noting that *Fischerella* NIES-4106 seems to have a fully sequenced cluster, but the cluster is missing genes and this might reflect a loss of the FaRLiP phenotype. The second column marks strains which have been proven to synthesize chlorophyll *f* (deep red) or are otherwise associated with red-shifted chlorophylls in non-axenic cultures (red).

Fragments appear to contain sufficient phylogenetic information to be assigned to large clades. These include (from bottom to top, in color blocks): Heterocyst-forming cyanobacteria, Chroococcales, Pleurocapsales, *Halomicronema* and *Synechococcus* PCC 7335. The polyphyletic sequences at the top are likely associated with Oscillatoriales.

This polyphyly could be an indication that the FaRLiP cluster is an ancient innovation. Even when only full sequences are considered (Figure 3), the high level of sequence divergence results in long branches. The Oscillatoriales branch out early from the cyanobacterial species tree (12). Vertical descent or early HGT of *apcE2* would fit early-branching strains having highly divergent, early-branching sequences. However, there also remains the possibility that some of these divergent sequences belong to an *Aphanocapsa* strain. *Aphanocapsa* strains have been found to undergo far-red acclimation, but there is little sequence data available, and no *apcE2* sequences (13,14).

Included in this phylogeny are fragments from the JGI metagenome archive (labelled JGI) and from the NCBI sequence read archive (labelled SRA). Multiple alignment: MUSCLE with Seaview. Misassembled sequences were removed, and very similar sequences from the same environments were collated into a consensus. Phylogeny: RaxML on the Cipres webserver, HPC2 on XSEDE, 100 bootstrap. Branches under 40 bootstrap were collapsed. Tree illustrated with iTOL and Inkscape.

| **Locus Tag** | **Genome ID** | **Metagenome Name** | **Notes** |
| --- | --- | --- | --- |
| SL_9KL_010_SEDDRAFT_100086822 | 3300000365 | Alkali sediment microbial communities from Tanatar-3 Soda Lake, Russia - 9KL_010_SED (*) (MER-FS) (assembled) | Originally mislabeled location in the JGI database (‘Soda Lake, California, USA’).  (Gerard Mujzer, University of Amsterdam, personal communication). (15) |
| SL_1KL_011_SEDDRAFT_100074853 | 3300000362 | Alkaline sediment microbial communities from Cock Soda Lake, Russia, USA - 1KL_011_SED (*) (MER-FS) (assembled) | Originally mislabeled location in the JGI database (‘Soda Lake, California, USA’).  (Gerard Mujzer, University of Amsterdam, personal communication). (15) |
| Ga0099817_11128282 | 3300008031 | Coral microbial communities from Puerto Morelos, Mexico - Siderastrea C B metatranscriptome (Eukaryote Community Metatranscriptome) (*) (MER-FS) (assembled) | Samples taken from a water depth of 2m. Control sample (~28°C tank, 25 days).  (Viridiana Avila Magana, Penn State University, personal communication) |
| Ga0099818_11279663 | 3300008016 | Coral microbial communities from Puerto Morelos, Mexico - Siderastrea C C metatranscriptome (Eukaryote Community Metatranscriptome) (*) (MER-FS) (assembled) | Samples taken from a water depth of 2m. Control sample (~28°C tank, 25 days).  (Viridiana Avila Magana, Penn State University, personal communication) |
| Ga0099819_10575014 | 3300008006 | Coral microbial communities from Puerto Morelos, Mexico - Siderastrea T A metatranscriptome (Eukaryote Community Metatranscriptome) (*) (MER-FS) (assembled) | Samples taken from a water depth of 2m. Heat-shock sample (~28°C tank for 16 days, then 34°C for 9 days).  (Viridiana Avila Magana, Penn State University, personal communication) |
| Ga0099820_10680134 | 3300008033 | Coral microbial communities from Puerto Morelos, Mexico - Siderastrea T B metatranscriptome (Eukaryote Community Metatranscriptome) (*) (MER-FS) (assembled) | Samples taken from a water depth of 2m. Heat-shock sample (~28°C tank for 16 days, then 34°C for 9 days).  (Viridiana Avila Magana, Penn State University, personal communication) |
| Ga0099821_10420704 | 3300008030 | Coral microbial communities from Puerto Morelos, Mexico - Siderastrea T C metatranscriptome (Eukaryote Community Metatranscriptome) (*) (MER-FS) (assembled) | Samples taken from a water depth of 2m. Heat-shock sample (~28°C tank for 16 days, then 34°C for 9 days).  (Viridiana Avila Magana, Penn State University, personal communication) |
| Ga0049069_10025324 | 3300003753 | Cyanobacterial communities from the Joint Genome Institute, California, USA - FECB-21 (*) (MER-FS) (assembled) | Samples described as ‘host-associated organisms recalcitrant to axenic growth’ in JGI GOLD. Dataset produced under U.S. Department of Energy Contract No. DE-AC02-05CH11231. Thanks to Matthias Hess (UC Davis). |
| JGI12140J13697_10001814 | 3300001230 | Estuarine microbial mat communities from Elkhorn Slough, Moss Landing, CA, USA - CR2B Metatranscriptome (Metagenome Metatranscriptome) (*) (MER-FS) (assembled) | Sample generation and sequencing for this dataset was supported by the LLNL Biofuels Scientific Focus Area SCW1039, and Join Genome Institute Community Sequencing Program award #701 to Jennifer Pett-Ridge (Lawrence Livermore National Laboratory). |
| JGI12140J13697_10001845 | 3300001230 | Estuarine microbial mat communities from Elkhorn Slough, Moss Landing, CA, USA - CR2B Metatranscriptome (Metagenome Metatranscriptome) (*) (MER-FS) (assembled) | Sample generation and sequencing for this dataset was supported by the LLNL Biofuels Scientific Focus Area SCW1039, and Join Genome Institute Community Sequencing Program award #701 to Jennifer Pett-Ridge (Lawrence Livermore National Laboratory). |
| Ga0224722_100015528 | 3300021518 | Freshwater sediment microbial communities from Lake Towuti, South Sulawesi, Indonesia - Balambano_FR1_MetaG (*) (MER-FS) (assembled) | Permission kindly granted by Rachel Simister and Sean Crowe (University of British Columbia). |
| Ga0224722_10071622 | 3300021518 | Freshwater sediment microbial communities from Lake Towuti, South Sulawesi, Indonesia - Balambano_FR1_MetaG (*) (MER-FS) (assembled) | Idem. |
| Ga0224723_100008721 | 3300021517 | Freshwater sediment microbial communities from Lake Towuti, South Sulawesi, Indonesia - Balambano_FR2_MetaG (*) (MER-FS) (assembled) | Idem. |
| Ga0224723_100019429 | 3300021517 | Freshwater sediment microbial communities from Lake Towuti, South Sulawesi, Indonesia - Balambano_FR2_MetaG (*) (MER-FS) (assembled) | Idem. |
| Ga0224723_100028118 | 3300021517 | Freshwater sediment microbial communities from Lake Towuti, South Sulawesi, Indonesia - Balambano_FR2_MetaG (*) (MER-FS) (assembled) | Idem. |
| Ga0073934_100102832 | 3300006865 | Hot spring sediment bacterial and archaeal communities from British Columbia, Canada, to study Microbial Dark Matter (Phase II) - Larsen N4 metaG (*) (MER-FS) (assembled) | Permission kindly granted by Tanja Woyke (DOE JGI). |
| Ga0209172_100216242 | 3300025310 | Hot spring sediment bacterial and archaeal communities from British Columbia, Canada, to study Microbial Dark Matter (Phase II) - Larsen N4 metaG (SPAdes) (*) (MER-FS) (assembled) | Idem. |
| Ga0008833_10013643 | 3300005143 | Hypersaline microbial mat communities from Hot Lake, Washington, USA - Hot Lake Consortium UCC-LE (*) (MER-FS) (assembled) | Identical to all other sequences labelled ‘Hot Lake, Washington, USA’ or ‘*Leptolyngbya* sp. HL7711’. Permission kindly granted by Stephen Lindermann (Purdue University). |
| Ga0008834_100047625 | 3300005142 | Hypersaline microbial mat communities from Hot Lake, Washington, USA - Hot Lake Consortium UCC-L28 (*) (MER-FS) (assembled) | Idem. |
| Ga0008835_10062125 | 3300005140 | Hypersaline microbial mat communities from Hot Lake, Washington, USA - Hot Lake Consortium UCC-RE (Hybrid Assembly) (*) (MER-FS) (assembled) | Idem. |
| Ga0008836_1004173 | 3300005141 | Hypersaline microbial mat communities from Hot Lake, Washington, USA - Hot Lake Consortium UCC-R28 (Hybrid Assembly) (*) (MER-FS) (assembled) | Idem. |
| Ga0008837_1005023 | 3300005412 | Hypersaline microbial mat communities from Hot Lake, Washington, USA - Hot Lake Consortium UCC-SLE (*) (MER-FS) (assembled) | Idem. |
| Ga0008838_1006333 | 3300005139 | Hypersaline microbial mat communities from Hot Lake, Washington, USA - Hot Lake Consortium UCC-SL28 (Hybrid Assembly) (*) (MER-FS) (assembled) | Idem. |
| Ga0076929_10036047 | 3300009069 | Hypersaline microbial mat communities from Hot Lake, Washington, USA - Hot Lake Consortium UCC-SL28 (version 3) (*) (MER-FS) (assembled) | Idem. |
| Ga0076930_10010587 | 3300005996 | Hypersaline microbial mat communities from Hot Lake, Washington, USA - Hot Lake Consortium UCC-RE (version 3) (*) (MER-FS) (assembled) | Idem. |
| Ga0079973_10071934 | 3300005717 | Hypersaline microbial mat communities from Hot Lake, Washington, USA - Hot Lake Consortium UCC-R28 (version 2) (*) (MER-FS) (assembled) | Idem. |
| Ga0079974_10008174 | 3300006016 | Hypersaline microbial mat communities from Hot Lake, Washington, USA - Hot Lake Consortium UCC-R28 (version 3) (*) (MER-FS) (assembled) | Idem. |
| Ga0081520_10003544 | 3300005974 | Hypersaline microbial mat communities from Hot Lake, Washington, USA - Section #1 (SPADES assembly) (*) (MER-FS) (assembled) | Idem. |
| Ga0081526_10008029 | 3300005973 | Hypersaline microbial mat communities from Hot Lake, Washington, USA - Section #2 (SPADES assembly) (*) (MER-FS) (assembled) | Idem. |
| Ga0081528_10007147 | 3300005976 | Hypersaline microbial mat communities from Hot Lake, Washington, USA - Section #4 (SPADES assembly) (*) (MER-FS) (assembled) | Idem. |
| Ga0081535_10003866 | 3300005957 | Hypersaline microbial mat communities from Hot Lake, Washington, USA - Section #3 (SPADES assembly) (*) (MER-FS) (assembled) | Idem. |
| Ga0208112_10013435 | 3300025675 | Hypersaline microbial mat communities from Hot Lake, Washington, USA - Hot Lake Consortium UCC-LE (SPAdes) (*) (MER-FS) (assembled) | Idem. |
| Ga0208218_100008413 | 3300025384 | Hypersaline microbial mat communities from Hot Lake, Washington, USA - Section #3 (SPAdes) (*) (MER-FS) (assembled) | Idem. |
| Ga0208353_10019121 | 3300025348 | Hypersaline microbial mat communities from Hot Lake, Washington, USA - Section #2 (SPAdes) (*) (MER-FS) (assembled) | Idem. |
| Ga0208354_100030421 | 3300025364 | Hypersaline microbial mat communities from Hot Lake, Washington, USA - Section #4 (SPAdes) (*) (MER-FS) (assembled) | Idem. |
| Ga0208477_1002203 | 3300025354 | Hypersaline microbial mat communities from Hot Lake, Washington, USA - Section #1 (SPAdes) (*) (MER-FS) (assembled) | Idem. |
| Ga0208623_10010735 | 3300026561 | Hypersaline microbial mat communities from Hot Lake, Washington, USA - Hot Lake Consortium UCC-L28 (SPAdes) (*) (MER-FS) (assembled) | Idem. |
| Ga0208979_10015635 | 3300025347 | Hypersaline microbial mat communities from Hot Lake, Washington, USA - Hot Lake Consortium UCC-SLE (SPAdes) (*) (MER-FS) (assembled) | Idem. |
| JGI12278J13113_10004504 | 3300000986 | Hypersaline microbial mat communities from Hot Lake, Washington, USA - Section #2 (*) (MER-FS) (assembled) | Idem. |
| JGI12322J13274_10001084 | 3300001112 | Hypersaline microbial mat communities from Hot Lake, Washington, USA - Section #3 (*) (MER-FS) (assembled) | Idem. |
| JGI12437J12857_10004502 | 3300000910 | Hypersaline microbial mat communities from Hot Lake, Washington, USA - Section #4 (*) (MER-FS) (assembled) | Idem. |
| JGI12519J13273_10002314 | 3300001101 | Hypersaline microbial mat communities from Hot Lake, Washington, USA - Section #1 (*) (MER-FS) (assembled) | Idem. |
| Ga0115029_100209814 | 3300009415 | Marine algal microbial communities from Sidmouth, United Kingdom - Sidmouth_Asex1 metaG (*) (MER-FS) (assembled) | Intertidal. Exposed by low tides ~5h/day. (Susan Brawley, University of Maine, personal communication). Dataset  produced as part of the *Porphyra umbilicalis* genome project (16) under US Department of Energy Contract DE-AC02-05CH11231. |
| Ga0115030_10002645 | 3300009072 | Marine algal microbial communities from Sidmouth, United Kingdom - Sidmouth_Male1 metaG (*) (MER-FS) (assembled) | Intertidal. Exposed by low tides ~5h/day. (Susan Brawley, University of Maine, personal communication). Dataset  produced as part of the *Porphyra umbilicalis* genome project (16) under US Department of Energy Contract DE-AC02-05CH11231. |
| Ga0197796_10322 | 3300019532 | Sorted cell/s from microbial mat in Hot Lake, Washington, United States? *Leptolyngbya* sp. HL7711_P1F1 JGI 000148CP-K16 (*) (MER-FS) (assembled) | Identical to all other sequences labelled ‘Hot Lake, Washington, USA‘ or ‘*Leptolyngbya* sp. HL7711’. Permission kindly granted by Stephen Lindermann (Purdue University). |
| Ga0197797_11854 | 3300019527 | Sorted cell/s from microbial mat in Hot Lake, Washington, United States? *Leptolyngbya* sp. HL7711_P1F1 JGI 000148CP-G19 (*) (MER-FS) (assembled) | Idem. |
| Ga0197798_101035 | 3300019529 | Sorted cell/s from microbial mat in Hot Lake, Washington, United States? *Leptolyngbya* sp. HL7711_P1F1 JGI 000148CP-H11 (*) (MER-FS) (assembled) | Idem. |
| Ga0197800_12224 | 3300019531 | Sorted cell/s from microbial mat in Hot Lake, Washington, United States? *Leptolyngbya* sp. HL7711_P1F1 JGI 000148CP-G14 (*) (MER-FS) (assembled) | Idem. |
| **Table S3.** Far-red ApcE2 homologues recovered from the metagenomic data available at the JGI/MER database. Some sequences represent identical copies of others in the same environments. Far-red photosynthesis appears to be present in a diverse range of environments. Many thanks to all the researchers responsible for individual datasets, as well as the JGI database, for allowing us to use this data. These sequence data were produced by the US Department of Energy Joint Genome Institute http://www.jgi.doe.gov/ in collaboration with the user community. The Joint Genome Institute provides these data in good faith, but makes no warranty, expressed or implied, nor assumes any legal liability or responsibility for any purpose for which the data are used. | | | |

| **Locus Tag** | **Genome ID** | **Metagenome Name** | **Notes** |
| --- | --- | --- | --- |
| Ga0182874_119577481 | 3300015215 | Freshwater microbial mat communities from Canadian High Arctic Lake, Ward Hunt Island, Canada - Sample WHb | Sample contained also bacteriochlorophyll, another far-red light asorbing pigment (17). Permission kindly granted by Adrien Vigneron, Newcastle University. |
| TB FS06_10DRAFT_3112356611 | 3300000233 | Groundwater microbial communities from subsurface biofilms in sulfidic aquifer in Frasassi Gorge, Italy, sample from two redox zones- FS06_10 | Permission kindly granted by Jennifer Macalady (Penn State University). |
| Ga0073934_3033804231 | 3300006865 | Hot spring sediment of bacterial and archaeal communities from British Columbia, Canada. Study on Microbial Dark Matter (Phase II) - Larsen N4 metaG |  |
| Ga0272446 100105726 | 3300028735 | Hot spring microbial mat communities from Yellowstone National Park, United States - YNP-CB-006-1 | Full gene* |
| Ga0137716 1000016852 | 3300010938 | Sediment microbial community from Chocolate Pots hot springs, Yellowstone National Park, Wyoming, USA. Combined Assembly of Gp0156111, Gp0156114, Gp0156117 | Full gene*. Oscillatoriales cyanobacteria represent a significant part of the sample  (18). |
| Ga0073899_3000783441 | 3300009540 | Active sludge microbial communities from Klosterneuburg, Austria, studying microevolution and ecology of nitrifiers - Klosterneuburg WWTP active sludge metagenome KNB5-Ph |  |
| Ga0215184_11874491 | 3300022595 | Metatranscriptome of coral microbial communities from Popa Island, Bocas del Toro, Panama - APAL T2 (Eukaryote Community Metatranscriptome) |  |
| Ga0103786_11384971 | 3300009348 | Microbial communities of thrombolites from Highborne Cay, Bahamas - Zone2_total_RNA |  |
| Ga0115029_10122191 | 3300009415 | Marine algal microbial communities from Sidmouth, United Kingdom - Sidmouth_Asex1 metaG | Dataset  produced as part of the *Porphyra umbilicalis* genome project (16) under US Department of Energy Contract DE-AC02-05CH11231. |
| Ga0105045_302980741 | 3300007517 | Freshwater microbial communities from Lake Fryxell liftoff mats and glacier meltwater in Antarctica - MAT-02 (megahit assembly) | Permission kindly granted by Joan Slonczewski, Kenyon College. |
| Ga0115031_307918911 | 3300009439 | Marine algal microbial communities from Maine, USA - Maine_Asex4_metaG | Dataset  produced as part of the *Porphyra umbilicalis* genome project (16) under US Department of Energy Contract DE-AC02-05CH11231. |
| Ga0115031_307414901 | 3300009439 | Marine algal microbial communities from Maine, USA - Maine_Asex4_metaG | Dataset  produced as part of the *Porphyra umbilicalis* genome project (16) under US Department of Energy Contract DE-AC02-05CH11231. |
| Ga0099817_13998551 | 3300008031 | Coral microbial communities from Puerto Morelos, Mexico - Siderastrea C B metatranscriptome (Eukaryote Community Metatranscriptome) |  |
| Ga0103784_10000125 | 3300008568 | Microbial communities of thrombolites from Highborne Cay, Bahamas - Zone1_total_RNA | Similar sample (19) |
| Ga0181613_10063651 | 3300014149 | *In-situ* water column microbial community from the vent pool of Chocolate Pots hot spring, Yellowstone National Park, Wyoming, USA - CP Vent Pool | (18) |
| SRR3280822.83895.2 | SRR3280822 | Characterization of microbial communities in Lake Clifton, Western Australia using whole shotgun metagenomics | (20) |
| SRR3280822.94895.1 | SRR3280822 | Characterization of microbial communities in Lake Clifton, Western Australia using whole shotgun metagenomics | (20) |
| SRR3311156.90036 | SRR3311156 | Metagenomic study of Alchichica microbialites, Mexico | (21) |
| SRR3401492.67650.2 | SRR3401492 | Metagenome from marine plastic debris: Sample Station: STN-15; Sample: 15b, Pacific Ocean | (22) |
| SRR3311145.98565.1 | SRR3311145 | Metagenomic study of Alchichica microbialites, Mexico | (21) |
| SRR3280822.44382.1 | SRR3280822 | Characterization of microbial communities in Lake Clifton, Western Australia using whole shotgun metagenomics | (20) |
| SRR405102.173542117.2 | SRR405102 | Baltic Sea site KBA sample SWE 02_21m |  |
| SRR2394720.69348.1 | SRR2394720 | Phylogenetic and Functional Substrate Specificity for Endolithic Microbial Communities from the Atacama Desert, Chile | (23) |
| SRA: Stromatolites, Mexico (SRR3310984.13984.2, SRR3310984.13984.1, SRR3311145.25056.1) | SRR3310984, SRR3311145 | Metagenomic study of Alchichica microbialites, Mexico | Consensus of multiple sequences. (21) |
| SRA: Pacific Garbage Patch 1 (SRR3401480.48706.1, SRR3401476.31320.2, SRR3401489.21847.1) | SRR3401480, SRR3401476, SRR3401489 | Metagenome from marine plastic debris: Sample Station: STN-15; Sample: 15b/ Sample Station: STN-11; Sample: 11b / Sample Station: STN-14; Sample: 14a. Pacific Ocean | Consensus of multiple sequences.  (22) |
| **Table S4.** *apcE2* fragments (unless otherwise noted) recovered from the JGI metagenomic database (above the double line) or from the Sequence Read Archive (SRA) at the NCBI (below the double line). The identification of these fragments as *apcE2* was confirmed through phylogeny and reverse-BLAST-searching (blasting the fragment against the whole NCBI nr database). *This additional dataset includes full sequences that are not present in Figure 3, as they were uploaded by their authors after the creation of the Figure 3 phylogeny. | | | |

| **Species** | **Strain** | **Protein sequence** |
| --- | --- | --- |
| *Calothrix parasitica* | NIES-267 | WP_096658610.1 |
| *Calothrix sp.* | NIES-3974 | WP_096620787.1 |
| *Calothrix sp.* | PCC 7507 | WP_015126587.1 |
| *Chlorogloeopsis fritchii* | PCC 6912 / 9212 | WP_016873423.1 |
| Cyanobacterium | TDX16 | OWY64183.1 |
| *Chroococcidiopsis sp.* | CCALA 051 | WP_106544528.1 |
| *Chroococcidiopsis thermalis* | PCC 7203 | WP_015153116.1 |
| *Cyanosarcina cf. burmensis* | CCALA 770 | WP_106218458.1 |
| *Fischerella major* | NIES-592 | OKH11132.1 |
| *Fischerella sp.* | JSC-11 | EHC19220.1 |
| *Fischerella sp.* | NIES-3754 | WP_062246790.1 |
| *Fischerella sp.* | NIES-4106 | WP_096680239.1 |
| *Fischerella sp.* | PCC 9605 | WP_026734727.1 |
| *Halomicronema hongdechloris* | C2206 | WP_080806386.1 |
| *Hydrococcus rivularis* | NIES-593 | WP_073600233.1 |
| *Leptolyngbya sp.* | JSC-1 | WP_051926079.1 |
| *Mastigocoleus testarum* | BC008 | WP_036265308.1 |
| Oscillatoriales cyanobacterium | JSC-12 | WP_009769103.1 |
| *Pleurocapsa sp.* | CCALA 161 | WP_106235916.1 |
| *Pleurocapsa minor* | PCC 7327 | WP_015143544.1 |
| *Synechococcus sp.* | PCC 7335 | WP_006455341.1 |
| *Fischerella muscicola* | CCMEE 5330 |  |
| *Fischerella muscicola* | PCC 7414, CCMEE 5323 |  |
| *Fischerella thermalis* | CCMEE 5205, CCMEE 5268 |  |
| *Fischerella thermalis* | CCMEE 5194 |  |
| *Fischerella thermalis* | CCMEE 5318 |  |
| *Fischerella thermalis* | CCMEE 5201 |  |
| *Fischerella thermalis* | CCMEE 5196, CCMEE 5198 |  |
| *Fischerella thermalis* | CCMEE 5282 |  |
| *Fischerella thermalis* | CCMEE 5328 |  |
| *Fischerella thermalis* | CCMEE 5208 |  |
| *Fischerella thermalis* | CCMEE 5273, WC213, WC246, WC249, WC341, WC344, WC439, WC442, WC527, WC538, WC542, WC558, WC559, WC1110 |  |
| *Fischerella thermalis* | WC246 |  |
| *Fischerella thermalis* | WC114, WC119, WC157, WC245 |  |
| *Fischerella thermalis* | BR2B |  |
| **Table S5.** Homologues of the far-red phycobilisome-core linker ApcE2. The sequences above the triple line were used to build the alignment in Figure 1 and find the extended VIPEDV motif associated with non-covalent binding of phycocyanobilin. Below the line there are additional *Fischerella* sequences with at least 95% identity to each other. Identical sequences are collated. | | |

|  | **North** | **Central** | **South** |
| --- | --- | --- | --- |
| **Non-lithifying** | N/A (0) | 0 *Pleurocapsa*-like  0 *Halomicronema*-like (23.6G) | N/A (0) |
| **Pustular** | N/A (0) | 7 *Pleurocapsa*-like  39 *Halomicronema*-like (28.4G) | N/A (0) |
| **Smooth** | 0 *Pleurocapsa*-like  0 *Halomicronema*-like (15.6G) | 0 *Pleurocapsa*-like  3 *Halomicronema*-like (11.8G) | N/A (0) |
| **Colloform** | 7 *Pleurocapsa*-like  0 *Halomicronema*-like (7.8G) | N/A (0) | 3 *Pleurocapsa*-like  3 *Halomicronema*-like (27.3G) |
| **Table S6.** Fragments of far-red *apcE2* in metagenomic unassembled data from stromatolites in Spaven Province, Shark Bay, Australia (project PRJNA429237). The columns represent sampling areas. The rows separate stromatolite types (with non-lithifying mats added for comparison). Numbers show how many far-red sequences were found via BLAST (homology determined through phylogenetic trees as described above). Numbers in parentheses represent the data available (in gigabases). There might be a pattern between stromatolite types and the lineages of far-red cyanobacteria that inhabit them. For example, *Halomicronema-*like sequences were found in all stromatolite types, but *Pleurocapsa*-like sequences were more common in samples from the seaward colloform stromatolites, as opposed to the more shoreward types. This could be a statistical artefact; however, it represents a starting point for further investigations. | | | |

| **Amplicon name** | **Related sequences** | **Amplicon name, GenBank** | **Gene** | **GenBank ID** |
| --- | --- | --- | --- | --- |
| Beach rock, environmental | *Pleurocapsa* | Br_env_ApcE2 | *apcE2* | MK465692 |
| Beach rock 2 | *Pleurocapsa* | Br2_ApcE2 | *apcE2* | MK465693 |
| Beach rock 4 and 5.2 | *Pleurocapsa* | Br4+5.2_ApcE2* | *apcE2* | MK465694 |
| Beach rock 5.1 | *Halomicronema* | Br5.1_ApcE2 | *apcE2* | MK465695 |
| *Halomicronema* Clifton | *Halomicronema* | Halo_Apce2 | *apcE2* | MK465696 |
| Beach rock 2’ | *Pleurocapsa* | Br2\'_16S | 16S rRNA | MK465697 |
| Beach rock 4’ | *Pleurocapsa* | Br4\'_16S | 16S rRNA | MK465698 |
| Beach rock 5.1’ | *Acaryochloris* | Br5.1\'_16S | 16S rRNA | MK465699 |
| Beach rock 5.2’ | *Acaryochloris* | Br5.2\'_16S | 16S rRNA | MK465700 |
| *Halomicronema* Clifton, 16S | *Halomicronema* | Halo_16S | 16S rRNA | MK465701 |
| **Table S7.** Gene fragments (*apcE2* and 16S rRNA) recovered in this study from beach rock and thrombolite samples. For beach rock, numbers mark different cultures (e.g. beach rock 2 vs beach rock 4) while decimals mark different sequences recovered from the same culture (e.g. beach rock 5.1, 5.2). Thrombolites are associated with the samples labelled ‘*Halomicronema* Clifton’. *The sequence was recovered from beach rock 4 and 5. | | | | |

**Supplementary Computational Citations**

We thank the people behind the SearchSRA project (24) for providing the open-access search of this immense repository, as well as the people who developed the original pieces of software that were later assembled into SearchSRA (25-28).

**Supplementary References**

(1) Wang J, Xia F, Zeleke J, Zou B, Rhee S, Quan Z. An improved protocol with a highly degenerate primer targeting copper-containing membrane-bound monooxygenase genes for community analysis of methane-and ammonia-oxidizing bacteria. FEMS Microbiol Ecol. 2017; 93(3): fiw244.

(2) Nübel U, Garcia-Pichel F, Muyzer G. PCR primers to amplify 16S rRNA genes from cyanobacteria. Appl Environ Microbiol. 1997; 63(8): 3327-3332.

(3) Ferris MJ, Muyzer G, Ward DM. Denaturing gradient gel electrophoresis profiles of 16S rRNA-defined populations inhabiting a hot spring microbial mat community. Appl Environ Microbiol. 1996; 62(2): 340-346.

(4) Rose TM. CODEHOP-mediated PCR–a powerful technique for the identification and characterization of viral genomes. Virol J. 2005; 2(1): 20.

(5) Lefever S, Pattyn F, Hellemans J, Vandesompele J. Single-nucleotide polymorphisms and other mismatches reduce performance of quantitative PCR assays. Clin Chem. 2013; 59(10): 1470-1480.

(6) Schirrmeister BE, de Vos JM, Antonelli A, Bagheri HC. Evolution of multicellularity coincided with increased diversification of cyanobacteria and the Great Oxidation Event. Proc Natl Acad Sci U S A. 2013; 110(5): 1791-1796.

(7) Dvořák P, Casamatta DA, Poulíčková A, Hašler P, Ondřej V, Sanges R. *Synechococcus*: 3 billion years of global dominance. Mol Ecol. 2014; 23(22): 5538-5551.

(8) Sánchez-Baracaldo P. Origin of marine planktonic cyanobacteria. Sci Rep. 2015; 5: 17418.

(9) Liu L, Chen X, Zhang Y, Zhou B. Characterization, structure and function of linker polypeptides in phycobilisomes of cyanobacteria and red algae: an overview. Biochim Biophys Acta. 2005; 1708(2): 133-142.

(10) Ponce-Toledo RI, Deschamps P, López-García P, Zivanovic Y, Benzerara K, Moreira D. An early-branching freshwater cyanobacterium at the origin of plastids. Curr Biol. 2017; 27(3): 386-391.

(11) Bailey TL, Boden M, Buske FA, Frith M, Grant CE, Clementi L, et al. MEME SUITE: tools for motif discovery and searching. Nucleic Acids Res. 2009; 37: W202-W208.

(12) Schirrmeister BE, Antonelli A, Bagheri HC. The origin of multicellularity in cyanobacteria. BCM Evol Biol. 2011; 11(1): 45.

(13) Behrendt L, Brejnrod A, Schliep M, Sørensen SJ, Larkum AW, Kühl M. Chlorophyll *f*-driven photosynthesis in a cavernous cyanobacterium. ISME J. 2015; 9: 2108-2111.

(14) Miyashita H, Ohkubo S, Komatsu H, Sorimachi Y, Fukayama D, Fujinuma D, et al. Discovery of chlorophyll *d* in *Acaryochloris marina* and chlorophyll *f* in a unicellular cyanobacterium, strain KC1, isolated from Lake Biwa. J Phys Chem Biophys. 2014; 4: 149.

(15) Vavourakis CD, Andrei A, Mehrshad M, Ghai R, Sorokin DY, Muyzer G. A metagenomics roadmap to the uncultured genome diversity in hypersaline soda lake sediments. Microbiome. 2018; 6(1): 168.

(16) Brawley SH, Blouin NA, Ficko-Blean E, Wheeler GL, Lohr M, Goodson HV, et al. Insights into the red algae and eukaryotic evolution from the genome of *Porphyra umbilicalis* (Bangiophyceae, Rhodophyta). Proc Natl Acad Sci U S A. 2017; 114(31): E6361-E6370.

(17) Vigneron A, Perrine Cruaud VM, Martineau M, Culley AI, Lovejoy C, Vincent WF. Multiple strategies for light-harvesting, photoprotection, and carbon flow in high latitude microbial mats. Front Microbiol. 2018; 9: 2881.

(18) Fortney NW, He S, Converse BJ, Boyd ES, Roden EE. Investigating the composition and metabolic potential of microbial communities in Chocolate Pots Hot Springs. Front Microbiol. 2018; 9: 2075.

(19) Louyakis AS, Mobberley JM, Vitek BE, Visscher PT, Hagan PD, Reid RP, et al. A study of the microbial spatial heterogeneity of Bahamian thrombolites using molecular, biochemical, and stable isotope analyses. Astrobiology. 2017; 17(5): 413-430.

(20) Warden JG, Casaburi G, Omelon CR, Bennett PC, Breecker DO, Foster JS. Characterization of microbial mat microbiomes in the modern thrombolite ecosystem of Lake Clifton, Western Australia using shotgun metagenomics. Front Microbiol. 2016; 7: 1064.

(21) Saghaï A, Zivanovic Y, Moreira D, Benzerara K, Bertolino P, Ragon M, et al. Comparative metagenomics unveils functions and genome features of microbialite‐associated communities along a depth gradient. Environ Microbiol. 2016; 18(12): 4990-5004.

(22) Bryant JA, Clemente TM, Viviani DA, Fong AA, Thomas KA, Kemp P, et al. Diversity and activity of communities inhabiting plastic debris in the North Pacific Gyre. mSystems. 2016; 1(3): e00024-16.

(23) Crits-Christoph A, Robinson CK, Ma B, Ravel J, Wierzchos J, Ascaso C, et al. Phylogenetic and functional substrate specificity for endolithic microbial communities in hyper-arid environments. Front Microbiol. 2016; 7: 301.

(24) Levi K, Rynge M, Abeysinghe E, Edwards RA. Searching the sequence read archive using Jetstream and Wrangler. in PEARC '18: Proceedings of the Practice and Experience on Advanced Research Computing, Pittsburgh, PA, USA, July 22–26 (ACM, New York, 2018).

(25) Stewart CA, Cockerill T, Foster I, Hancock DY, Merchant N, Skidmore E, et al. Jetstream: a self-provisioned, scalable science and engineering cloud environment. in Proceedings of the 2015 XSEDE Conference: Scientific Advancements Enabled by Enhanced Cyberinfrastructure: ACM; 2015.

(26) Torres PJ, Edwards RA, McNair KA. PARTIE: a partition engine to separate metagenomic and amplicon projects in the Sequence Read Archive. Bioinformatics. 2017; 33(15): 2389-2391.

(27) Towns J, Cockerill T, Dahan M, Foster I, Gaither K, Grimshaw A, et al. XSEDE: accelerating scientific discoverys. Computing in Science & Engineering. 2014; 16(5): 62-74.

(28) Buchfink B, Xie C, Huson DH. Fast and sensitive protein alignment using DIAMOND. Nat Methods. 2015; 12(1): 59-60.
